# An Updated Review of Tetrodotoxin and Its Peculiarities

**DOI:** 10.3390/md20010047

**Published:** 2022-01-03

**Authors:** Panagiota Katikou, Cengiz Gokbulut, Ali Rıza Kosker, Mònica Campàs, Fatih Ozogul

**Affiliations:** 1 Ministry of Rural Development and Food, Directorate of Research, Innovation and Education, Hapsa & Karatasou 1, 54626 Thessaloniki, Greece; 2 Department of Pharmacology, Faculty of Medicine, Balikesir University, Balikesir 10145, Turkey; cgokbulut@gmail.com; 3 Department of Seafood Processing Technology, Faculty of Fisheries, Cukurova University, Adana 01330, Turkey; akosker@cu.edu.tr; 4 IRTA, Ctra Poble Nou km 5.5, 43540 Sant Carles de la Ràpita, Spain; monica.campas@irta.cat

**Keywords:** marine toxins, tetrodotoxin (TTX), public health, edible marine organisms, pufferfish

## Abstract

Tetrodotoxin (TTX) is a crystalline, weakly basic, colorless organic substance and is one of the most potent marine toxins known. Although TTX was first isolated from pufferfish, it has been found in numerous other marine organisms and a few terrestrial species. Moreover, tetrodotoxication is still an important health problem today, as TTX has no known antidote. TTX poisonings were most commonly reported from Japan, Thailand, and China, but today the risk of TTX poisoning is spreading around the world. Recent studies have shown that TTX-containing fish are being found in other regions of the Pacific and in the Indian Ocean, as well as the Mediterranean Sea. This review aims to summarize pertinent information available to date on the structure, origin, distribution, mechanism of action of TTX and analytical methods used for the detection of TTX, as well as on TTX-containing organisms, symptoms of TTX poisoning, and incidence worldwide.

## 1. Introduction

Tetrodotoxin (TTX) is one of the most potent natural marine toxins, which acts by selectively blocking the action potentials of voltage-gated Na+ channels along nerves, skeletal and cardiac muscle membranes, without changing the resting membrane potentials. TTX was named after the Tetraodontidae pufferfish family, from which it was initially isolated and is considered the most lethal toxin found in the marine environment [1,2]. TTX is both water soluble and heat stable, therefore not being destroyed by heat processing; on the contrary, it rather increases its toxic effect, whereas there is no known antidote for TTX to date [3,4]. Despite its recognized poisonous, or sometimes lethal effects when ingested by humans at high doses (lethal doses range from 1.5–2.0 mg TTX, equal to blood level 9 ng/mL), when administered at much lower levels, TTX exhibits therapeutic properties, so far mainly targeted to treating cancer-related, neuropathic and/or visceral pain [5].

**Figure 1:**
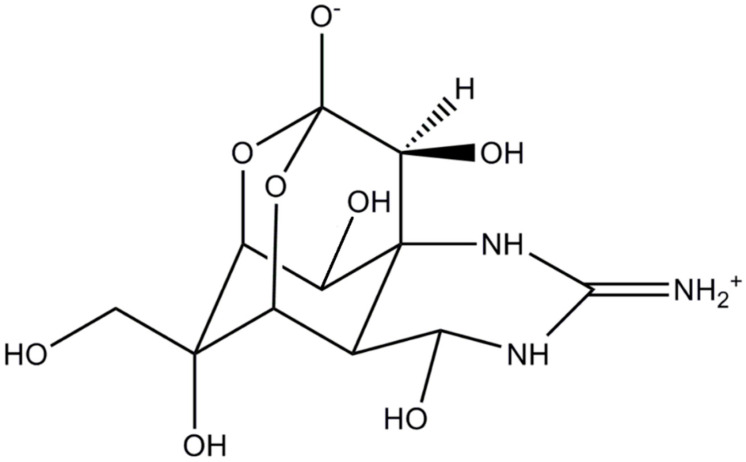
Structure of tetrodotoxin.

TTX is a crystalline, weak basic, colorless substance with a molecular formula of C_11_H_17_O_8_N_3_ (Figure 1). At least 30 structural analogues have been described to date, with varying degrees of toxicity. Depending on structure, these are classified into three groups: hemilactal, lactone, and 4,9-anhydro types, altogether referred to as tetrodotoxins (TTXs). TTXs are found in various, taxonomically diverse, groups of animals, dwelling in both terrestrial and aquatic (marine, freshwater, and brackish) environments [6,7,8]. TTX is believed to originate from bacteria belonging to the phylum Proteobacteria, comprising *Pseudomonas*, *Pseudoalteromonas*, and *Vibrio*; however, several other bacterial phyla (Actinobacteria, Bacterioides, Firmicutes, Proteobacteria) are occasionally reported as potential TTX sources [1,9]. Such TTX-producing bacteria (i.e., genera *Vibrio*, *Pseudomonas*, *Aeromonas*, *Alteromonas*, *Nocardiopsis*, *Bacillus*, *Shewanella*, *Roseobacter*) have been found in the subcutaneous mucus, ovaries, and the gastrointestinal tract of several aquatic species [3,10,11,12], while certain evidence exists the literature suggesting their association to specific dinoflagellate blooms, such as *Alexandrium tamarense* or *Prorocentrum cordatum* [13,14,15].

TTX has been responsible for numerous—occasionally fatal—human intoxication incidents and typically linked to pufferfish consumption, especially in countries of the Far East (particularly Japan), where they constitute a delicacy known as “fugu” [3]. Until the beginning of the 21st century, TTX was commonly found in tropical waters and was not being reported, or yet perceived as a potential hazard, in temperate areas, such as the Mediterranean Sea and Europe. Starting from 2003, however, a known TTX-vector, the pufferfish species *Lagocephalus sceleratus*, has been increasingly recorded in eastern Mediterranean coasts, owing to its invasion through the Suez Canal (the so-called Lessepsian migration). By 2007, the species had managed to establish its presence in these habitats, gradually expanding towards Israel, Lebanon, Turkey, Cyprus, and Greece [16,17,18,19,20] and later in the rest of the Mediterranean (Italy, Croatia, Malta, Libya, Algeria, and Tunisia), finally reaching Spain by mid-2014 [21,22,23]. 

Meanwhile, again, in 2007, the first human TTX-poisoning in Europe was also reported, attributed to the consumption of a TTX contaminated gastropod, the trumpet shell *Charonia lampas*, initially originating from the south of Portugal, but purchased in Malaga, Spain [24,25]. Later, in 2015, evidence on the occurrence of TTXs in European bivalve mollusks started to appear in the literature. So far, TTXs in shellfish have been reported in a number of European countries (UK, Greece, Netherlands, Portugal, Spain, Italy, France), with this presence detected in samples dating as early as 2006 and in a variety of species, including bivalve mollusks (mussels, oysters, venus clams) and gastropods (*Gibbula umbilicalis*, *Monodonta lineata*, and *C. lampas*) [7,14,26,27,28,29,30,31,32,33,34], but no further human TTX-intoxications were associated with these aquatic organisms. Similarly, there are reports on the presence of TTXs in several bivalve mollusks species from other temperate areas of the world, including New Zealand, China and Japan [8,35,36,37,38], with no relevant intoxications in humans. On the contrary, there are several reports of human poisoning in these “non-traditional” areas related to ingestion of TTX-containing pufferfish, mostly *L. sceleratus*, specifically in Israel, Lebanon, Palestine (Gaza strip), Turkey, Cyprus, and Greece, with at least three lethal cases in Turkey, and a suspected case of TTX poisoning associated with octopus’ consumption in Malta [17,39,40,41,42,43,44,45,46]. Expectably though, there is potentially significant underreporting of intoxication cases, especially as regards documentation in scientific literature, given that most incidents are generally only clinically diagnosed, without laboratory confirmation of TTX presence, while they are commonly reported in the local daily press. Consequently, important information may also be missed due to language barriers.

The observed increase of TTXs incidences, in terms of both presence in edible marine organisms and human poisoning cases, in countries where they were previously uncommon, has raised concerns about their management from a legislative point of view. So far, Japan remains the only country which has set an official regulatory limit for TTXs at 10 MU/g, equivalent to 2 mg TTX/kg pufferfish tissue [47,48]. The European Union (EU), despite the aforementioned records, has not yet established a maximum permitted level (MPL) for TTXs content in seafood, and this toxin group is not regularly monitored. In fact, as regards TTXs, the current EU legislation only foresees that fishery products derived from poisonous fish of the family Tetraodontidae must not be placed on the market, whereas similar regulatory requirements exist in other non-EU Mediterranean countries, such as Turkey and Egypt [18,49,50,51]. However, as an initial response to the concerns raised by the presence of TTXs in bivalve mollusks and gastropods, the European Commission requested the European Food Safety Authority (EFSA) Panel on “Contaminants in the Food Chain” to deliver a scientific opinion as regards the “risks related to the presence of TTXs in marine bivalves and gastropods”. The opinion was issued in early 2017, proposing a provisional concentration below 44 μg TTX equivalents/kg shellfish meat, which was considered not to result in adverse effects in humans [12]. Nevertheless, the opinion recognized several shortcomings related to availability of epidemiological data and of validated analysis methods, which highlighted the requirement for more solid evidence in order to proceed towards adopting a legislative MPL in the future [47]. 

The present review summarizes the latest available information concerning the origin and sources of TTXs, their worldwide distribution in seafood, their mode of action and their effects, studied both in vivo and in vitro, details on their toxicity in humans, experimental animal species or in vitro, treatment protocols for the management of human TTX-poisoning cases, their pharmacological properties and beneficial effects for human health when exploited in treatment protocols for certain human diseases, and, finally, the recent developments on quantification and screening methods for TTXs, for the purpose of unravelling the risks derived from this toxin group and contributing towards establishing management regimes for human health protection.

## 2. Origin and Sources of TTX

Understanding the origin of TTX in edible marine organisms and the mechanisms by which this toxin accumulates in the food chain is essential for effective risk assessment and management. Unfortunately, to date the data available on elucidating these processes remain controversial. The most popular theories suggest that TTXs are either produced by symbiotic bacteria (endogenous route), or that they are exogenously accumulated through the diet [8]. An intermediate explanation entailing the involvement of microalgae, specifically dinoflagellates, bearing the symbiotic TTX-produced bacteria has also been suggested; the TTX-bearing microalgae are subsequently consumed by aquatic organisms during their feeding processes, resulting in TTXs accumulation in the latter [15].

As regards the bacterial symbiosis hypothesis, although the evidence is contradictory, a wide diversity of bacterial species and strains has been associated to TTX production. At least 150 bacterial strains have been reported as TTX-producers, with the *Vibrio* genus being the major representative, comprising more than 30% of the total TTX-producing strains. For instance, *V. alginolyticus* strains present in the aquatic animals’ microflora have been associated with TTX production. Strains belonging to the *Bacillus* genus, on the other hand, constitute approximately another 15% of the isolated TTX-producers, while the genera *Pseudomonas*, *Alteromonas*, *Aeromonas*, *Streptomyces* and *Roseobacter* comprise up to 7% of TTX-producing bacteria, with the remaining genera only represented by maybe a single strain each [9]. According to other researchers, *Vibrio* is still considered the most common bacterial TTX-producing genus, followed by *Bacillus*, *Pseudomonas*, *Actinomyces*, and *Micrococcus* [52]. Nevertheless, no evidence of TTX production in cultured bacterial strains isolated from the blue-ringed octopus *Hapalochlaena* sp., the sea slug *Pleurobranchaea maculata*, and the gastropod *Nassarius semiplicatus* were obtained by means of advanced analytical techniques, such as liquid chromatography coupled to mass spectrometry (LC-MS), despite the wide diversity of bacterial species encountered in these species [53]. 

To explain this controversy, an overestimation regarding the number of TTX-producing bacteria, attributed to analytical constraints, has been suggested [8]. It is also possible, however, that certain TTX producing organisms are not easily culturable under standard laboratory conditions or the strains isolated were not able to produce TTX ex situ, despite their genetic ability for TTX biosynthesis [53]. The latter is further supported by the reported existence of a *Bacillus* bacterial strain (*Bacillus* sp. 1839) exhibiting a stable TTX production constant after five years of cultivation under laboratory conditions since its isolation [54], despite the fact that loss of production with further inoculations is commonly observed with many cultured strains, indicating potential contamination by the starting material [8]. 

A further finding in support of the connection between TTX presence and toxin-producing marine bacteria was the first two individuals of the marine nemertean species *Cephalothrix simula* recently discovered in the UK [55]. *C. simula* originates from the Pacific Ocean and is long known to be associated with high TTX levels. 16S gene sequencing of the *C. simula* microbiome conducted to establish its taxonomic assignment confirmed the prevalence of numerous bacterial genera associated to TTX production, such as *Alteromonas*, *Vibrio*, and *Pseudomonas*. Liquid chromatography coupled to tandem mass spectrometry (LC-MS/MS) analysis of the nemertean tissue revealed the presence of multiple TTX analogues, additional to the dominant parent TTX, with the total TTXs reaching a concentration as high as 54 μg TTX/g tissue. Additionally, two individual bacterial strains, the first belonging to the species *Pseudomonas luteola* isolated from the toxic *C. simula* specimen, and the second belonging to *Vibrio alginolyticus* originating from a non-toxic native *Tubulanus annulatus* nemertean, were cultured at low temperature (22^o^C). Their extracts were analyzed by LC-MS/MS and were found to contain the parent TTX, at levels of 93 ng and 88 ng TTX per liter of culture, respectively [52]. Certain works supporting an entirely endogenous origin of TTX even support that, in the case of some pufferfish species, where TTX plays a defense mechanism role, extant bacteria are not involved anymore in TTX biosynthesis, despite the initial bacterial origin of their biosynthetic genes [56]. Notably though, TTX is not present in exclusively freshwater-living fauna, not returning to aqueous systems for breeding reasons [57], which may also point to an association of TTX production with marine bacteria. 

The involvement of microalgal species in the bacterial symbiosis theory has also been suggested. Significant TTX amounts were detected in cultured cells of the dinoflagellate *A. tamarense*, where a link to endocellular bacteria was suggested [13]. Furthermore, the existence of a link between the presence of TTX-producing bacteria and *Prorocentrum cordatum (P. minimum)* dinoflagellates was suspected when TTX was found in shellfish harvested from Greek coastal waters during a concurrent *P. cordatum* bloom [14]. This observation was later strengthened by studies involving these particular shellfish specimens and selected reference strains of *P. cordatum* originating from Ecuador and Johor Strait, between Singapore and Malaysia [15]. Extracts obtained by these *P. cordatum* strains contained two new compounds (*m/z* 265 and *m/z* 308) with similar ion pattern and C9-base to TTX analogues, which were related to the presence of two TTX-related bacteria, *Roseobacter* and *Vibrio* sp. The new compounds exhibited TTX-like effects on sodium current inhibition (INa), with a direct relation observed between INa and the concentration of the *m/z* 265 compound. Symbiotic production was, thus, considered as a potential explanation for the fact that *P. cordatum* is not always toxic [14]. As such, subsequent studies in England could not establish a link between *P. cordatum* and TTXs during the years 2013–2016, despite the common detection of *P. cordatum* at variable cell densities in areas along the southern coast of England [55]. Similarly, it was not possible to correlate the presence of *P. cordatum* with that of TTX in shellfish harvested from French coastal areas [30]. 

Nevertheless, some scientific evidence strongly indicate the exogenous toxification of aquatic organisms as a valid theory [56], that is: (1) the remarkable individual and regional variations in pufferfish toxicity, (2) TTX accumulation by the trumpet shell *Charonia sauliae* through ingestion of toxic starfish, and (3) TTX absence in artificially cultured pufferfish *Takifugu rubripes* and *T. niphobles* when fed non-toxic diets, in contrast to their ability for efficient accumulation following oral TTX administration. In this context, it is suggested that aquatic organisms, such as pufferfish, do not synthesize TTX, but acquire it instead from other toxic organisms bearing bioconcentrated TTX through the food chain with marine bacteria being at the start [58]. Indeed, it has been experimentally shown that egg plates of toxic *Planocera multitentaculata* flatworms contributed to the toxification of *T. niphobles* at various stages of its life [59,60]. Similarly, studies on TTX micro-distribution in New Zealand *Paphies australis* clams found that, among all tested tissues, siphons were those with the highest TTX contents, while immunohistochemistry revealed that TTX was present in the siphons’ outer cells, as well as in the digestive system, foot, and gill tissue, that is the organs related to feeding. These findings further support the exogenous origin hypothesis, at least where *P. australis* is concerned [61]. Further studies on TTX depuration using the same species held in captivity and fed a toxin-free diet for 150 days, at intervals of three to 15 days, showed that the highest TTX concentrations were found in siphons, during the whole depuration study. Where digestive glands are concerned, though, only low TTX contents were measured at the start of the experiment which rapidly depurated with only traces remaining after 21 days [37]. 

To date, the exact biosynthetic pathways and genes for TTX production are still unclear [47]. Nevertheless, it is believed that TTX biosynthetic pathways of terrestrial and marine animals differ, as most TTX analogues are only detected in either terrestrial or marine organisms, with only few exceptions of analogues found in both [62]. Recent studies by Ueyama et al. [63] isolated seven novel spiro-bicyclic-guanidino compounds (compounds 2–8) from the pufferfish species *Tetraodon biocellatus*; the carbon skeleton and relative configuration of six of them (compounds 2–5 and 7–8) was the same as in TTX. These seven compounds were considered to be biosynthetic intermediates of TTX for marine environment organisms and, more specifically, assumed to be precursors of 5,6,11-trideoxyTTX, as none of them had been detected in toxic terrestrial species, such as newts. Further LC-MS analyses were in support of this assumption, confirming that two of these compounds (2–3) are widely distributed in numerous different TTX-bearing marine organisms, specifically five pufferfish species (*Tetraodon biocellatus*, *Takifugu. chrysops*, *Takifugu flavipterus*, *Arothron manilensis*, *Chelonodon patoca*), one crab species (*Atergatis floridus*), and one octopus’ species (*Hapalochlaena lunulata*), as well as *Planocerid* sp. Flatworms. Moreover, it was proposed that production of marine TTXs and related compounds, including compounds 2–8, was due to marine microorganisms, subsequently accumulating in TTX-containing marine animals. However, further screening would be required to confirm the presence of the above TTX-related compounds in marine microorganisms, as well as bivalve mollusks and gastropods, in order to elucidate the exact biosynthetic pathway to TTX, combined with attempts to locate the precursors of compound 7 and TTX-related genes [8,63].

As regards TTXs presence in marine bivalves or edible gastropods, the existing limited evidence available so far indicate an exogenous source, with TTX accumulation in bivalves mainly linked to Gammaproteobacteria, especially *Vibrio* and *Pseudomonas* sp. Recent works [29,55] have correlated TTX presence in shellfish to the simultaneous presence of *Vibrio* and *Pseudomonas*, although no cultures of any TTX-related bacteria were possible to obtain from the samples analyzed. The assumption that bacteria or micro-algae are linked to TTX production is further supported by the seasonality of TTX in bivalves located mostly during warmer months, that is late spring and summer, in European countries [14,28,29,30,31,32,33,55], as well as New Zealand [37,64], indicating the involvement of TTX-producing microorganisms adapted to warmer water temperatures.

## 3. Occurrence and Distribution of TTXs in Potentially Edible Aquatic Organisms

The presence of TTXs in potentially edible aquatic organisms, especially pufferfish, has been long documented. The oldest records date back to the beginning of the 20th century, when Tahara and Hirata [65] isolated a toxin from the ovaries of fish belonging to the Tetraodontidae family, which was named Tetrodotoxin [66]. TTX was believed to exist exclusively in pufferfish, until the mid-1960s, when Mosher et al. [67] reported that the eggs of the California newt *Taricha torosa* contained TTX [67]. Since then, TTXs have been identified in a wide variety of aquatic animals, including bivalve mollusks, gastropods, and echinoderms, as well as other mollusks and crustaceans. This section attempts to summarize mostly relevant data of the last 20 years, with a special emphasis placed on the occurrence of TTXs in aquatic organisms found in sub-tropical and temperate regions not previously considered as endemic for TTXs and in species not traditionally perceived as TTX vectors. Some of these aquatic organisms may be rarely found in markets or are not considered as edible, per se. However, intoxications thereof are frequently reported due to accidental consumption, mostly related to recreational fishing or harvesting and consumption by local populations, unaware of potential restrictions imposed by the legislation or monitoring programs. 

### 3.1. TTXs in Pufferfish 

Pufferfish are the most notorious TTX source, responsible for countless human intoxication cases, especially in countries of the Indian Ocean or the Far East, such as Japan, where they have been historically consumed as a delicacy and have established a unique food culture associated with these organisms [68,69]. TTXs presence in pufferfish and associated human poisoning incidents around the world have been extensively reviewed by the works of Arakawa et al. [68], Guardone et al. [45], Tamele et al. [69], and Bédry et al. [43]. In the present work, TTXs occurrence and distribution in pufferfish is focused on specimens caught in sub-tropical or temperate areas which, until recently, were not considered as susceptible to TTX presence, such as countries around the Mediterranean Sea. Table 1 summarizes the relevant published data on pufferfish TTX maximum contents and their distribution among the individual tissues studied. In general, the highest TTXs concentrations are found in the gonads, liver, and gastrointestinal tract, followed by muscle and skin. The levels in all cases, however, are higher than those considered as safe for human consumption, considering either the Japanese safety limit of 10 mouse units (MU)/g, equivalent to 2000 μg TTX/kg pufferfish tissue (1 MU = 0.2 μg TTX), or the stricter provisional concentration of 44 μg TTX/kg shellfish meat indicated by the recent EFSA opinion on TTXs [47]. As the minimum lethal dose for humans is estimated to be approximately 10,000 MU, equivalent to a total ingestion of 2 mg TTX for a 50 kg human [18,58], it is evident by the TTXs concentrations reported that all these pufferfish species are dangerous for human consumption. In this context, marketing of pufferfish, namely those of the Tetraodontidae, Molidae, Diodontidae, and Canthigasteridae families, is prohibited by the European or national legislation of the countries affected [47,49,50,51,70]. Nevertheless, accidental pufferfish consumption by people unaware of the risks entailed has repeatedly occurred, resulting in severe or sometimes lethal human intoxication cases, as described in part 5 of the present review.

**Table 1:** Tetrodotoxins (TTXs) presence in pufferfish originating from non TTX-endemic areas. TTXs concentration refers to the sum of quantified TTX analogues. DG = digestive gland, WF = whole flesh, LIV = Liver, GON = Gonads, MUS = Muscle tissue (flesh), SK = Skin, GIT = Gastrointestinal tract, INT = Intestine; MBA = Mouse bioassay; ELISA = Enzyme-Linked Immunosorbent Assay; LC-MS = Liquid chromatography mass spectrometry; LC-MS/MS = Liquid chromatography tandem mass spectrometry; Q-TOF LC/MS = Quadrupole Time-of-Flight Liquid chromatography mass spectrometry. Concentrations marked in bold red font exceed the Japanese regulatory limit of 2000 μg TTX/kg.

Country	Sampling Year	Common Name	Species	Tissue	Maximum TTXs Concentration (µg TTX eq/kg)	Analysis Method Used for Quantification	Reference
Croatia	2014	Silver stripe blaasop (pufferfish)	*Lagocephalus sceleratus*	LIV	30,600	LC-MS/MS	[23]
				GON	48,700		
				MUS	800		
				SK	1500		
Greece	2007	Silver stripe blaasop (pufferfish)	*Lagocephalus sceleratus*	LIV	87,530	MBA	[18]
				GON	239,320		
				GIT	177,650		
				MUS	10,160		
				SK	6630		
				LIV	1,380,800	LC-MS/MS	[71]
				GON	8,248,510		
				GIT	478,430		
				MUS	58,440		
				SK	33,340		
	2017–2020	Silver stripe blaasop (pufferfish)	*Lagocephalus sceleratus*	LIV	312,950	LC-MS/MS	[72]
				GON	535,780		
				MUS	41,470		
				SK	35,050		
Spain	2014	Silver stripe blaasop (pufferfish)	*Lagocephalus sceleratus*	LIV	3080	LC-MS/MS	[22]
				GON	25,950		
				MUS	1010		
				SK	1650		
Portugal (São Miguel Island, Azores)	2013	Guinean puffer (pufferfish)	*Sphoeroides marmoratus*	LIV	765	LC-MS/MS	[73]
				GON	696,631		
				MUS	15,921		
Turkey	2012	Silver stripe blaasop (pufferfish)	*Lagocephalus sceleratus*	LIV	46,180 ± 2060	LC-MS/MS	[74]
				GON	52,070 ± 4600		
				INT	7,150 ± 1330		
				MUS	2,830 ± 920		
				SK	3,430 ± 1130		
	2017–2018	Silver stripe blaasop (pufferfish)	*Lagocephalus sceleratus*	LIV	13,480	ELISA	[75]
				GON	12,870		
				INT	11,740		
				MUS	8320		
				SK	6540		
	2015–2016	Yellow spotted pufferfish	*Torquigener flavimaculosus*	LIV	106,800 ± 5560	LC-MS/MS	[76]
				GON	100,710 ± 6360		
				INT	86,300 ± 840		
				MUS	86,070 ± 2050		
				SK	139,880 ± 12,210		
	2015–2016	Suez puffer	*Lagocephalus suezensis*	LIV	1440 ± 30	Q-TOF LC/MS	[51]
				GON	2020 ± 80		
				INT	1910 ± 90		
				MUS	1440 ± 170		
				SK	3090 ± 280		

### 3.2. TTXs in Bivalve Mollusks

Worldwide, the presence of TTXs has been reported so far in 14 bivalve mollusk species harvested from 9 different countries (Table 2). The first record was in the digestive glands of Japanese scallops of the species *Mizuhopecten yessoensis* (previously *Patinopecten yessoensis*) with TTXs contents calculated at approximately 8000 μg/kg [77]. The next oldest records originate from New Zealand and refer to shellfish collected during 2001–2003, but tested much later as frozen archived samples [64]. As regards TTXs occurrence in Europe, the first record dates back to digestive glands of *Mytilus galloprovincialis* mussels and *Venus verrucosa* clams harvested in 2006 and 2008, respectively, in Greece and retrospectively analyzed as retained official monitoring samples [14]. A number of further reports on TTXs presence in bivalve mollusks harvested between 2013 and 2019 from six more countries, that is, England, the Netherlands, China, Italy, Spain, and France, gradually appeared in the literature [26,27,28,29,30,31,32,33,34,35,55,78], along with further TTX records from additional species in New Zealand [38,64,79,80]. So far, among the highest TTXs concentrations detected in bivalve mollusks were those of 1600 μg/kg in Greenshell^TM^ mussels (*Perna canaliculus*) [64] and 800 μg/kg in “pipi” clams (*Paphies australis*) from New Zealand [79], 541 μg/kg in Mediterranean mussels (*M. galloprovincialis*) from Italy [32], and 253 μg/kg in Pacific oysters (*Magallana gigas*) from England and the Netherlands [28,55]. Nevertheless, TTXs contents exceeded the provisional limit of 44 μg/kg proposed by EFSA in many other cases, as indicated in Table 2. 

**Table 2:** Tetrodotoxins (TTXs) presence in bivalve mollusks. TTXs concentration refers to the sum of quantified TTX analogues. Species names have been updated to comply with the currently accepted taxonomy—names in brackets denote the species name originally reported in the relevant reference. * Values recalculated from MU/g assuming 1 MU = 0.2 μg TTX unless otherwise specified in the relevant reference; DG = digestive gland, WF = whole flesh; MBA = Mouse bioassay; LC-MS = Liquid chromatography mass spectrometry; LC-MS/MS = Liquid chromatography tandem mass spectrometry. Concentrations marked in bold red font exceed the EFSA-proposed provisional limit of 44 μg TTX eq/kg.

Country	Sampling Year	Common Name	Species	Tissue	Maximum TTXs Concentration (µg TTX eq/kg)	Analysis Method Used for Quantification	Reference
China	2013–2014	Short-necked clam	*Ruditapes philippinarum*	WF	2.22	LC-MS/MS	[35]
		Chinese razor clam	*Sinonovacula constricta*	WF	16		
		Mussels	*Mytilus edulis*	WF	2.74		
		Hard-shelled mussel	*Mytilus coruscus*	WF	4.39		
England	2013	Pacific oysters	*Magallana gigas (Crassostrea gigas)*	WF	57.4	LC-MS/MS	[81]
	2014			WF	137		
	2014	Mussels	*Mytilus edulis*	WF	44		
	2015	Pacific oysters	*Magallana gigas (Crassostrea gigas)*	WF	253	LC-MS/MS	[55]
		Mussels	*Mytilus edulis*	WF	73		
	2016	Native oysters	*Ostrea edulis*	WF	~80		
		Hard clams	*Mercenaria mercenaria*	WF	72		
	2019	Pacific oysters	*Magallana gigas (Crassostrea gigas)*	WF	75	LC-MS/MS	[78]
				DG	242		
	2020	Clams	*Cerastoderma edule*	WF	6.88	LC-MS/MS	[26]
France	2018	Mussels	*Mytilus edulis*/ *galloprovincialis*	WF	11.2	LC-MS/MS	[30]
	2018	Pacific oysters	*Magallana gigas (Crassostrea gigas)*	WF	12.2	LC-MS/MS	[33]
	2018	Clams	*Ruditapes philippinarum*	WF	8.3		
	2019	Pacific oysters	*Magallana gigas (Crassostrea gigas)*	WF	32		
Greece	2006	Mussels	*Mytilus galloprovincialis*	DG	77.4	LC-MS/MS	[14]
	2008			DG	86.2		
	2008	Venus clams	*Venus verrucosa*	DG	194.7		
	2009	Mussels	*Mytilus galloprovincialis*	DG	71.3		
	2012			DG	222.9		
	2012			WF	193.6		
	2014			WF	47.0		
Italy	2015	Mussels	*Mytilus galloprovincialis*	WF	3.6	LC-MS/MS	[34]
	2016			WF	6.4		
	2017			WF	5.1		
	2017	Mussels	*Mytilus galloprovincialis*	WF	541	LC-MS/MS	[32]
	2018			WF	216		
	2018	Mussels	*Mytilus galloprovincialis*	WF	~10	LC-MS/MS	[31]
	2019			WF	76		
	2019			DG	196		
Japan	1993	Scallop	*Mizuhopecten yessoensis (Patinopecten yessoensis)*	DG	~8000 *	MBA	[77]
	2017	Scallop	*Mizuhopecten yessoensis (Patinopecten yessoensis)*	WF	7.3	LC-MS/MS	[36]
				DG	134.2		
New Zealand	2011	NZ rock oyster	*Saccostrea glomerata*	WF	140	LC-MS	[80]
	2011	Pacific oysters	*Magallana gigas (Crassostrea gigas)*	WF	80		
	2011	Pipi clams	*Paphies australis*	WF	160		
	2012			WF	120		
	2014	Pipi clams	*Paphies australis*	WF	800	LC-MS/MS	[79]
	2017	Pipi clams	*Paphies australis*	WF	220	LC-MS/MS	[64]
	2001–2003	Shellfish	(unknown)	WF	19	LC-MS/MS	[64]
	2007–2009	Shellfish	(unknown)	WF	21		
	2016	Greenshell^TM^ mussels	*Perna canaliculus*	WF	1600		
	2017	Pipi clams	*Paphies australis*	WF	470		
	2017	Greenshell^TM^ mussels	*Perna canaliculus*	WF	160	LC-MS/MS	[82]
Spain	2017	Cockles	(unknown)	WF	2.3	LC-MS/MS	[29]
	2017	Oysters	(unknown)	WF	0.9		
The Netherlands	2015	Mussels	*Mytilus edulis*	WF	33	LC-MS/MS	[12]
	2015	Pacific oysters	*Magallana gigas (Crassostrea gigas)*	WF	124	LC-MS/MS	[28]
	2016	Mussels	*Mytilus edulis*	WF	101		
	2016	Pacific oysters	*Magallana gigas (Crassostrea gigas)*	WF	253		
	2017	Mussels	*Mytilus edulis*	WF	>20		
	2017	Pacific oysters	*Magallana gigas (Crassostrea gigas)*	WF	51		

### 3.3. TTXs in Gastropods and Echinoderms

Gastropods are the second most frequent source of TTX poisoning, after pufferfish [45], with numerous human intoxication cases appearing in the literature, mostly in Asian countries, such as Taiwan, Japan, China, and Vietnam, where TTXs have traditionally occurred and where such aquatic organisms are a popular food [83]. Worldwide records of TTXs presence in gastropods responsible for human poisoning incidents have been thoroughly summarized by the works of Arakawa et al. [68], Biessy et al. [8], and Guardone et al. [45], while records in echinoderms are scarce. The present review attempts to summarize only the recent available data on recent TTXs occurrences in gastropod and echinoderm specimens originating particularly from non-endemic sub-tropical and temperate areas, which are presented in Table 3. 

The first documented presence of TTXs in gastropods from temperate areas was associated with a nonlethal food poisoning incident in Spain and involved the trumpet shell species *Charonia lampas* (formerly *Charonia lampas lampas*) harvested from the south coast of Portugal in 2007. The specimen’s digestive glands contained extremely high parent TTX and total TTXs levels, with reported concentrations of 315,000 and 1,319,000 μg/kg, respectively [25]. Further investigations in Portuguese and Moroccan gastropods revealed the presence of TTXs at quantifiable levels in three more species, *Steromphala umbilicalis* (formerly *Gibbula umbilicalis*), *Phorcus lineatus* (formerly *Monodonta lineata*), and *Onchidella celtica*, as well as in additional *C. lampas* specimens, some at high concentrations [7,73,84,85]. In general, it appears that higher TTXs contents are detected in the viscera of gastropods, potentially indicating a dietary route of accumulation; however, in some species, TTXs are exclusively detected in the flesh instead, which suggests the possible existence of alternative TTX binding mechanisms or sources [37].

At around the same period, the first detection of TTXs in the southern hemisphere occurred in New Zealand in 2009, which resulted from the investigation of poisoning incidents in dogs after consuming washed-up native grey side-gilled sea slugs, *Pleurobranchaea maculata*, in Auckland [86]. *P. maculata* was found to contain TTX at levels as high as 850,000 μg/kg [87]. This discovery triggered more intensive TTX testing in the years to follow, where TTXs were found in additional *P. maculata* specimens, as well as in bivalve mollusks (see Table 2) and gastropod and echinoderm species, such as *Arachnoides zelandiae* and *Lunella smaragda* (formerly *Turbo smaragdus*) [80,88].

**Table 3:** Tetrodotoxins (TTXs) presence in marine gastropods and echinoderms. TTXs concentration refers to the sum of quantified TTX analogues. Species names have been updated to comply with the currently accepted taxonomy—names in brackets denote the species name originally reported in the relevant reference; DG = digestive gland, WF = whole flesh, GON = Gonads, ST = Stomach, MUS = Muscle tissue (flesh), VIS = Viscera; MBA = Mouse bioassay; HPLC-FLD = High performance liquid chromatography with fluorescence detection; LC-MS = Liquid chromatography mass spectrometry; LC-MS/MS = Liquid chromatography tandem mass spectrometry. Concentrations marked in bold red font exceed the EFSA-proposed provisional limit of 44 μg TTX eq/kg.

Country	Sampling Year	Common Name	Species	Tissue	Maximum TTXs Concentration (µg TTX eq/kg)	Analysis Method Used for Quantification	Reference
France	2018	Whelk	*Buccinum undatum*	DG+GON+ST	7.4	LC-MS/MS	[30]
Morocco	2013	Sea slug	*Onchidella celtica*	WF	24	LC-MS/MS	[73]
New Zealand	2009	Sea slug	*Pleurobranchaea maculata*	WF	850,000	LC-MS/MS	[87]
	2010	Sand dollar	*Arachnoides zelandiae*	WF	250	LC-MS	[88]
	2010	Sea slug	*Pleurobranchaea maculata*	WF	1,414,000	LC-MS	[89]
	2011			WF	205,600		
	2011	Cat’s eye	*Lunella smaragda (Turbo smaragdus)*	WF	110	LC-MS	[80]
	2011	Sea slug	*Pleurobranchaea maculata*	WF	6		
	2012			WF	5.3		
Portugal	2007	Trumpet shell	*Charonia lampas (Charonia lampas lampas)*	DG	1,319,000	LC-MS	[25]
	2009	Sea snail	*Steromphala umbilicalis (Gibbula umbilicalis)*	WF	63.81	LC-MS/MS	[7]
	2010	Sea snail	*Phorcus lineatus (Monodonta lineata)*	WF	111.98		
	2010	Trumpet shell	*Charonia lampas*	WF	6.22		
	2010	Trumpet shell	*Charonia lampas*	MUS	66.6	LC-MS/MS	[84]
				VIS	22.4		
	2017	Trumpet shell	*Charonia lampas*	Edible MUS	119.3	LC-MS/MS	[85]
				Non-edible VIS	98,488.5		

### 3.4. TTXs in other Mollusks and Crustaceans

TTXs occurrence in crustaceans and other mollusks (excluding bivalves, gastropods, and echinoderms) appears scarce, and, to our knowledge, the recent available data have not been summarized. Records found in the literature on TTXs presence in these aquatic organisms are presented in Table 4. During the past decade, increased levels of TTXs have been reported in two blue-lined octopus species (*Hapalochlaena lunulata* and *H. fasciata*) in Taiwan and Japan [90,91,92]. The highest TTXs concentrations were detected in the posterior salivary glands, and it is believed that TTX in these species acts as a hunting and defense mechanism against predators [90]. These octopus species are not considered as edible; however, misidentification can result in severe morbidity and constitutes a significant risk from a food hygiene point of view [91]. In fact, the Taiwanese record is associated with a nonfatal human poisoning incident due to misidentification in the market and subsequent consumption [90]. 

**Table 4:** Tetrodotoxins (TTXs) presence in crustaceans and other mollusks (excluding bivalve mollusks, gastropods, and echinoderms). TTXs concentration refers to the sum of quantified TTX analogues. Species names have been updated to comply with the currently accepted taxonomy—names in brackets denote the species name originally reported in the relevant reference. * Values recalculated from MU/g assuming 1 MU = 0.2 μg TTX unless otherwise specified in the relevant reference; WF = whole flesh, WS = whole specimen, GON = gonads, PSG = posterior salivary gland, BM = buccal mass, OIO = Other internal organs, APP = appendages, VIS = viscera, STCON = Stomach contents, MUS = muscle tissue (flesh), SK = skin, ABD = abdomen, ARM = arms, CEP = cephalothorax; MBA = Mouse bioassay; LC-MS = Liquid chromatography mass spectrometry; LC-MS/MS = Liquid chromatography tandem mass spectrometry. Concentrations marked in bold red font exceed the EFSA-proposed provisional limit of 44 μg TTX eq/kg.

Country	Sampling Year	Common Name	Species	Tissue	Maximum TTXs Concentration (µg TTX eq/kg)	Analysis Method Used for Quantification	Reference
China	2013–2014	Horseshoe crab	*Carcinoscorpius rotundicauda*	WF	162	LC-MS/MS	[35]
Japan	2015	Greater blue-ringed octopus	*Hapalochlaena lunulata*	WS	13,232 *	MBA	[91]
	2016				17,907 *		
	2017				23,491 *		
	2019	Blue-lined octopus	*Hapalochlaena fasciata*	MUS+SK	12,900	LC-MS/MS	[92]
				GON	1980		
				PSG	287,000		
				BM	13,400		
				OIO	24,200		
	2018	Xanthid crab	*Zosimus aeneus*	APP	24,233	LC-MS/MS	[93]
				VIS	11,238		
	2019			APP	28,225		
				STCON	881		
New Zealand	2011	Seven-armed starfish	*Astrostole scabra*	WF	170	LC-MS	[80]
Portugal (São Miguel Island, Azores)	2013	Red velvet starfish	*Ophidiaster ophidianus*	WF	44,242	LC-MS/MS	[73]
Taiwan	2010	Blue-lined octopus	*Hapalochlaena fasciata*	WS	38,723	LC-MS/MS	[90]
				ARM	39,208		
				ABD	67,985		
				PSG	819,600		
				CEP	88,127		

Recent TTXs occurrences in other mollusks include two starfish species, *Astrostole scabra* and *Ophidiaster ophidianus*, from New Zealand and Portugal, respectively, with the latter specimen containing TTXs at levels as high as 44,242 μg/kg [73,80]. As regards crustaceans, two recent records were found in the literature. The first referred to a Chinese *Carcinoscorpius rotundicauda* horseshoe crab, containing much lower TTXs levels of 162 μg/kg [35], whereas the second reported TTX concentrations as high as 78.225 μg/kg in the appendages of *Zosimus aeneus* xanthid crabs harvested in Japan [93].

Finally, it should be noted that TTXs at concentrations up to 500 mg/kg have been reported in marine worms and flatworms. Although these species are not edible, they live inside other organisms, such as shellfish [52,94]. As such, their presence inside edible aquatic organisms could result in adverse health effects to consumers, constituting a potential risk requiring further investigation [8]. 

## 4. Mode of Action of TTX

The TTX molecule, with its unique chemical structure, blocks the passing of sodium ions through the cell membranes of nerve cells [95,96,97,98]. Voltage-gated sodium channels are essential ion channels for resting potential and neuronal excitability, which are crucial for the generation and propagation of action potentials in neurons [99]. 

**Figure 2:**
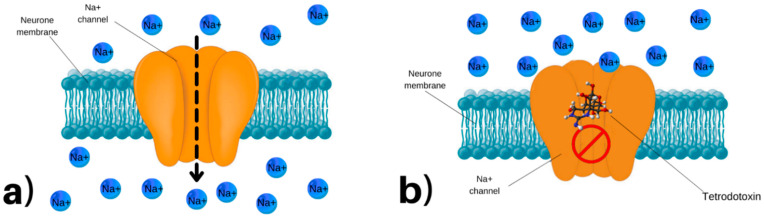
TTX action mechanism in voltage-gated Na^+^ channels of neuron cell. (**a**) Normal ion passing, (**b**) blocked by TTX.

Studies have shown that different tissues in different animals have different resistance to TTX (Figure 2) [97,100]. To date, at least nine different sodium channel isoforms have been identified in the mammalian nervous system [101,102]. Three of these, Na_v_1.5 (IC_50_ = 5.7 µM), Na_v_1.8 (IC_50_ = 60 µM), and Na_v_1.9 (IC_50_ = 40 µM), are resistant to TTX (IC_50_ = 5.7 µM). Other isoforms, predominantly expressed in skeletal muscle and nerve system, such as Na_v_1.1 (IC_50_ = 6 nM), Na_v_1.2 (IC_50_ = 18 nM), Na_v_1.3 (IC_50_ = 4 nM), Na_v_1.4 (IC_50_ = 25 nM), Na_v_1.6 (IC_50_ = 6 nM), and Na_v_1.9 (IC_50_ = 40 nM), are highly sensitive to TTX [102]. 

TTX is a potent neurotoxin well known for its ability to inhibit the voltage-sensitive sodium channel with potential primary blockade of the brainstem, somatic motor, sensory, and autonomic nerves [103]. Recent studies have determined that voltage-gated sodium channels not only play an important role in the normal electrophysiological activities of neurons but also have a close relationship with neurological diseases [101]. After the discovery of the blocking property of Na channels in nerve cells of the TTX molecule in the 1960s, the studies mostly shifted to the cellular and molecular mechanisms of TTX [98,104]. 

Due to this specific effect on nerve cells, TTX can be lethal to humans and many other animals. The effects of TTX and relevant poisoning incidents have a long history. The first Europeans to get seriously poisoned were Captain Cook and his naturalists, J.R. and G. Forster. They enjoyed a meal of pufferfish, survived, and fully described their symptoms [102,105,106]. Several case reports were known until 1941, when Fukuda and Tani provided a clinical grading system for TTX poisoning [107]. Clinical symptoms and findings of human TTX intoxications are discussed in detail in Section 5.2.

## 5. Toxicity of TTX 

### 5.1. Toxicity of TTX (in Human, Experimental Animals, and In Vitro)

Certain marine and terrestrial animals are known to be poisonous, as they contain considerable amounts of toxic compounds (e.g., alkaloids, steroids, terpenes, or other compounds) able to cause serious intoxication or fatality. Both these animals and their products are not expected to be consumed as food, and, as a consequence, relevant intoxication cases are generally considered as rare [108]. Pufferfish (fugu), although being known TTX vectors, constitute an exception to this rule, as they are widely consumed in some far eastern countries, especially in Japan, as a delicacy marine product [108]. 

The majority of cases associated with TTX intoxication and related fatalities by fugu occur mainly in Japan, China, and Taiwan, where pufferfish are traditionally consumed and considered a delicacy [24]. While, at the earlier part of the last century, there were about 100 deaths per year in Japan, more recent data indicate that there are about 40–50 intoxication cases per year, and about 10% of these result in death [58]. The likely reason for this dramatic reduction in cases and deaths is the rigorous training of fugu masters or cooks. Therefore, the majority of fugu poisoning cases today are a result of inexperienced fishermen or some people with low economic conditions consuming TTX-containing seafood in different far eastern countries, such as China and Bangladesh [109]. However, TTX poisoning has lately shown a rising trend since the threat of TTX-containing fish (e.g., pufferfish) is no longer limited to people in Asian countries but has spread to a wider geography by means of migration to the Pacific and Mediterranean relevant to the worldwide increase in temperature waters [17,18]. Moreover, in 2007, the first case of TTX poisoning in Malaga, Spain, by ingestion of a trumpet shellfish species collected in the southern Portuguese waters, indicated the arrival of the toxin in European coasts [25]. Due to the global spread of pufferfish and other TTX-bearing organisms and the increase in international aquaculture trade, it is possible that TTX poisoning cases will further increase [58].

**Figure 3:**
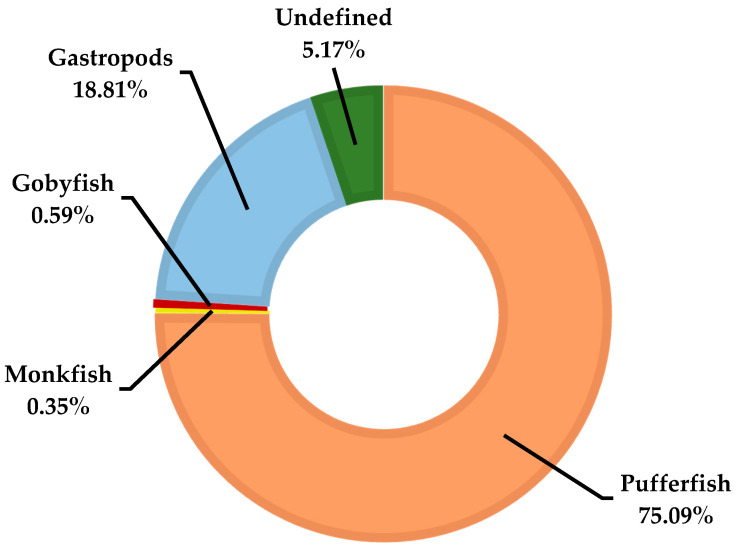
Marine species involved in TTX poisoning within the cases collected in this study.

The TTX concentration and its accumulation in the pufferfish body can greatly vary between species. Generally, in most species, the liver, ovary, and skin have the highest TTX concentrations, while muscle tissue and/or testis may be non-toxic or only contain traces of TTX, except for some species, such as *Chelonodon patoca*, *Lagocephalus lunaris*, and *L. sceleratus* [18,58]. Although the majority of TTX poisonings reported in the world are due to pufferfish consumption (Figure 3), several other marine organisms, such as the blue-ringed octopus, gobies, horseshoe crab, newts, frogs, starfish, and gastropods, may also produce and/or accumulate TTX, and their consumption may result in poisoning and death [110]. 

TTX is known as one of the most powerful and deadly toxins and about 1000 times more toxic to humans than cyanide [2,111]. Because of being a nonprotein toxin, TTX is heat stable; thus, cooking processes, including frying, boiling, baking, or stewing for hours, do not destroy or eliminate it [112]. Blocking of sodium ion channels by TTX, in turn, causes gastrointestinal and neurologic, as well as cardiac, failures, and, in many cases, death by respiratory paralysis in patients following oral exposure to the toxin [113]. Moreover, there is currently no effective antidote for TTX that can be used in the treatment of intoxications [104]. However, recovery can be achieved with early diagnosis and supportive treatment [114]. If the victim does not die of respiratory failure within 24 h, the patient could recover without any disorders [110].

The lethal potency of the TTX is 5000 to 6000 MU/mg [1 MU (mouse unit) is defined as the amount of toxin required to kill a 20 g male mouse within 30 min after intraperitoneal administration], and the minimum lethal dose (MLD) for humans is estimated to be approximately 10,000 MU (≈2 mg) [48], but this value can vary depending on age and health status of victims. Researchers investigated the toxicity of TTX in experimental animals, including mice, rats, and rabbits, to determine some toxicity parameters, which are summarized in Table 5. The oral median lethal dose (LD_50_) and No Observed Adverse Effect Level (NOAEL) values obtained in mice were 232 µg/kg and 75 µg/kg, respectively. On the other hand, Lago and co-workers reported that the LD_50_ values obtained in mice were 532 µg/kg for intragastric, 12.5 µg/kg for subcutaneous, and 10.7 µg/kg for intraperitoneal administrations [2]. In addition, the minimal lethal doses (MLD) of TTX in rabbits were calculated as 3.1 and 5.3 µg/kg, and the lethal doses (LD_100_) were calculated as 3.8 and 5.8, µg/kg, for intravenous and intramuscular administrations, respectively [115]. In the same study, it was also reported that TTX was found to be about fifty times less toxic to mice by the oral route compared to intraperitoneal administration, where clinical symptoms of TTX intoxication were considered in mice and rabbits. In addition, some pharmacokinetic and toxicity parameters were investigated in rats following oral administration of TTX pellets. Results indicated that LD_50_ of TTX was 517.43 μg/kg, while TTX reached a maximum plasma concentration (C_max_) at about 2 h, with an elimination half-life time (t_1/2_) of 3.23 ± 1.74 h after intragastric administration in rats at a dose of 100 μg/kg [116]. Thus, although there is variation in the parameters of TTX toxicity depending on the experimental model and the experimental animal species, these studies show that TTX is an extremely toxic compound for all mammalian species.

**Table 5:** Some toxicity parameters of TTX; ig: intragastric administration, im: intramuscular administration, ip: intraperitoneal administration, iv: intravenous administration, sc: subcutaneous administration.

Species	Route	Minimum LD	Median LD_50_	LD_100_	NOAEL	References
Human			2 mg (estimation for 50kg BW)			[48]
					13.33 μg/kg	[117]
Rats	oral		517.43 μg/kg			[116]
		10 µg/kg				[118]
Rabbit	im	5.3 µg/kg		5.8 µg/kg		[115]
	iv	3.1 µg/kg		3.8 µg/kg		
Mice	oral		232 µg/kg		75 µg/kg	[119]
	ip		10.7 µg/kg			[2]
	sc		12.5 µg/kg			
	ig		532 µg/kg			
	ip	40 µg/kg (for 4-CysTTX) and 860 µg/kg (for 4-GSTTX)				[120]
	ip		71 μg/kg (for 11-deoxyTTX)			[121]
	ip		10 μg/kg			[122]
	sc		16 μg/kg			
	oral		332 μg/kg			
	oral		619–700 mg/kg	1200 μg/kg		[123]
	ip		10.7 μg/kg			[115]
	sc		12.5 μg/kg			
	ig		532 μg/kg			

### 5.2. Clinical Symptoms and Findings of Human TTX Intoxications

According to the symptoms observed in human TTX intoxication cases, a clinical grading system has been developed (Table 6) consisting of four-degree classification for TTX poisoning in human [107], and this grading system is still used today. The grade or severity of TTX poisoning depends upon the amount of toxic product ingested, the time after ingestion of TTX, and the age and the health status of victims that affect the clinical symptoms or clinical symptoms of TTX poisoning [17]. Numerous cases of human TTX intoxication, caused by pufferfish and other marine products in different countries, have been reported in the literature; these are summarized in Table 7.

**Table 6:** Symptoms of TTX intoxication according to the clinical grading system developed by Fukuda and Tani [107].

Grade	Clinical Symptoms	Onset
I	Oral numbness and paresthesia, sometimes accompanied by gastrointestinal symptoms (nausea)	5–45 min
II	Numbness of face and other areas, advanced paresthesia, motor paralysis of extremities, incoordination, slurred speech, but still normal reflexes	10–60 min
III	Gross muscular incoordination, aphonia, dysphagia, dyspnea, cyanosis, drop in blood pressure, fixed/dilated pupils, precordial pain, but victims are still conscious	15 min to several hours
IV	Coma, severe respiratory failure and hypoxia, severe hypotension, bradycardia, cardiac arrhythmia, heart continues to pulsate for a short period	15 min to 24 h (death has been recorded 17 min after ingestion)

After ingestion of the product, symptoms begin within 5–45 min, due to rapid absorption of TTX from the gastrointestinal tract [122]; however, this could be delayed for 3 or more hours [112]. In cases of mild TTX poisoning, the symptoms start within 30 min to 6 h after ingestion, with full recovery usually in 24 h [124]. However, TTX intoxications can be lethal, and death can occur even within the first 20 min following ingestion of products with high TTX levels [125]. In severe poisoning cases, clinical symptoms, such as perioral numbness or paresthesia and stomachache, may appear within 5–15 min, followed by vomiting, which may appear 2 h after ingestion of seafood or products containing TTX. Experimental animal studies indicated that TTX can induce macroscopic changes in the gastrointestinal system, with ultrastructural effects involving the spleen, small, and large intestine in mice [126], which might explain the GI symptoms, such as nausea or vomiting, hyperemesis, abdominal pain, gastrointestinal motility disorders, hematemesis, and diarrhea, observed in human TTX intoxication cases [7,90,124,127,128].

The severity of symptoms depends on the ingestion level of TTX, and death may occur as fast as 17 min after ingestion in most critical cases due to respiratory failure and cardiovascular collapse [124]. Less commonly, death could occur as a result of profound hypotension [111] or other complications, such as hypertensive congestive heart failure in victims with hypertension [129] or aspiration pneumonia [130]. 

Respiratory failure is the most explicit and serious toxic effect of TTX poisoning and is usually the cause of death among victims [131,132]. Severe TTX intoxication, which is typically characterized by cyanosis, areflexia, apnea, and hypotension, often triggered second or third-degree AV block [102]. Cardiovascular symptoms of TTX poisoning are progressive hypotension, bradycardia, depressed AV node conduction, other arrhythmias, and even asystole. Severe cardiac failure sings, such as ventricular asystole or conduction block, could occur following ingestion of seafood or products containing extremely high levels TTX and as a late sign in fourth degree intoxicated patients [125].

Some investigators have reported hypotension as the cause of death in some animal models of TTX intoxications [133]. Severe hypotension is one of the typical findings of TTX poisoning that may be associated with bradycardia [134]. However, bradycardia is not the primary cause of hypotension, although it may contribute to some extent to a reduced arterial blood pressure, in severe TTX intoxication cases. Nevertheless, TTX-induced hypotension can be sufficiently explained by a reduced vasomotor tone rather than affecting the heart or the vasomotor center [102,122,134]. In contrast to these, hypertension has been noted in some human TTX intoxication cases of mild severity [17,124]. It seems likely that, in non-lethal exposure of TTX, hypertension could occur as a result of an exaggerated reaction to sympathetic stimuli [135]. The clinical symptoms of TTX intoxication (Table 1) are similar to those observed in poisonings caused by paralytic shellfish toxins (PST). PSTs constitute a variable mixture of tetrahydropurine marine toxins, with saxitoxin (STX) as their main representative, and may be found in organisms, such as bivalve mollusks, pufferfish, crabs, and gastropods, along with TTX [3,77]. Although the chemical structures of TTX and STX are considerably different, both toxins have similar modes of action and produce neuromuscular paralysis by affecting interaction with voltage-gated sodium channels [136]. Both toxins and their derivatives block ion conduction causing inhibition of action potentials, inducing respiratory depression, failure, and death [137].

Pathological findings from human TTX poisoning cases reported in the literature are limited, with pulmonary edema and generalized congestion of the viscera noted in some fatal cases [112,129,138]. Information in the literature with regard to routine hematological and biochemical parameters from human TTX poisoning case reports is limited. Routine hematological and biochemical parameters were evaluated in human TTX intoxication outbreaks in Bangladesh (11 cases) [109] and Israel (13 cases) [17]. These parameters were generally within the normal range in all victims with the exception of hypokalemia (one patient), and hypercapnia and creatine phosphokinase (CPK) elevation (two patients) in the Israel outbreak. Electrocardiograms (ECGs) and chest x-rays appeared normal in all patients, and the brain computer tomography (CT) performed in the two most severely poisoned patients was evaluated as normal [17]. In addition, in a Taiwanese outbreak (17 cases), abnormal liver biochemistry test results were noted in one of five victims tested [124].

The prognosis of TTX intoxication in human is generally favorable, if adequate supportive treatment can be administered before cardiopulmonary arrest occurs. As TTX can cause coma, areflexia, dilated pupils, or other common signs of brain injury early at the onset of poisoning, resuscitation should not be abandoned prematurely, even if these symptoms are present [139,140]. If a patient survives for more than 24 h, recovery is likely to occur if there are no complications with other life-threatening conditions [124,141,142].

### 5.3. Global Cases of TTX Intoxication in Humans

Human poisoning by marine toxins has been long occurring in human history, and poisoning cases have been recorded for thousands of years [143]. For instance, poisonous pufferfish, belonging to the Tetraodontidae family, were documented in the Ancient Chinese Pharmacopoeia compiled in 2800 BCE; thus, the warnings and recommendations associated with handling pufferfish were documented [143]. Similarly, the first Europeans to be poisoned from TTX were Captain Cook and his naturalists, J.R. and G. Forster. They ate pufferfish, survived, and described their precise symptoms, involving mostly clinical grade I and II manifestations (Table 6), such as experiencing weakness, dizziness, numbness, nausea, and shortage of breath [102,105,106].

The natural habitats of toxic marine species are generally the Pacific and Indian oceans, where tropical marine ecosystems are prominent. Societies living in these regions are familiar with poisonous fish and poisoning cases arising by the consumption of poisonous fish, as such incidents have been occurring in these regions for thousands of years. One of the toxins implicated in such poisoning events is TTX. Despite the fact that TTX was first isolated from pufferfish, it has been also found in other marine organisms and a few terrestrial species (e.g., newt and frog) [144]. However, pufferfish species remain the most common source of TTX poisoning (Figure 3).

**Figure 4:**
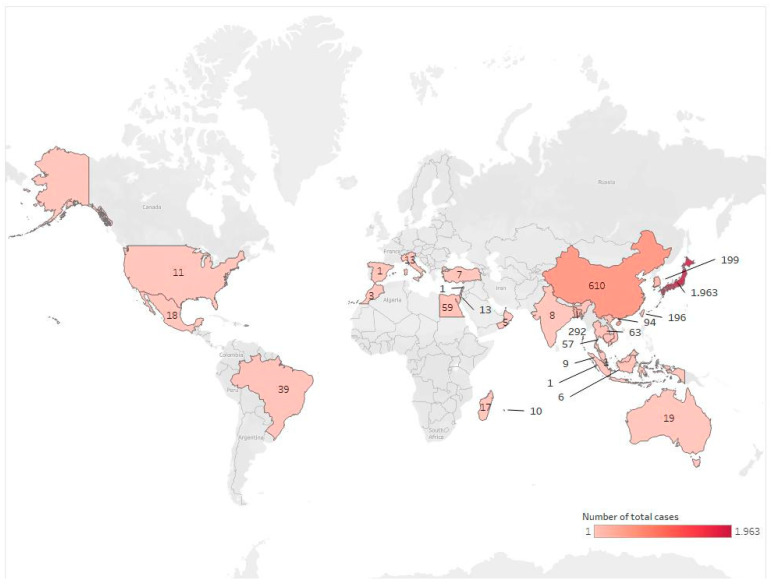
Regional distribution of the TTX poisoning cases collected in this study.

Expectably, TTX poisonings are most commonly reported from Japan (Table 7), as pufferfish consumption has an important place in traditional Japanese food culture, thus explaining why cases of poisoning have been recorded for so long and in such numbers (Figure 4) [68,110,145]. Death rates in Japan have gradually decreased following the extended studies on detecting toxic and non-toxic species aiming to ensure safe consumption, as well as due to the awareness-raising efforts undertaken in parallel with the spread of aquaculture [110]. Nevertheless, since people still cannot visually distinguish between toxic and non-toxic pufferfish or other TTX-containing organisms (mollusks, crabs, etc.), they cannot be adequately protected from poisoning; thus, poisoning rates cannot be reset [110]. Similarly, there have been many reported TTX poisoning cases from China [146,147], where poisonings are usually caused by marine species other than pufferfish. In Hong Kong, on the other hand, poisonings are generally seen among fishermen, as it is forbidden to sell pufferfish in the market, and only non-toxic cultured pufferfish prepared by licensed masters in restaurants and imported from Japan are allowed to be consumed [110,148]. In addition, many poisoning cases have also been reported in countries where pufferfish and other marine species containing TTX are native, such as Thailand [149,150], Taiwan [90,151,152], Cambodia [153], South Korea [154,155,156,157], Vietnam [130,158], Malaysia [159,160], Bangladesh [109,161,162,163], Indonesia [164], Singapore [165], and India [166].

Until recently, TTX was believed to be confined to East Asia, but recent studies have shown that fish containing the toxin have been found in other regions of the Pacific and the Indian Ocean, as well as the Mediterranean Sea [3]. There are also many poisoning cases reported from countries, such as Australia [167,168], New Caledonia [169], Brazil [170,171], Mexico [172], Oman [173], Madagascar [174], Reunion Island [175], Egypt [176], and Morocco [177].

TTX poisoning in the Mediterranean has emerged as a new phenomenon within the last two decades. Poisonous fish were not an issue of concern in Mediterranean countries until recently. In terms of TTX poisoning, among many species that entered the Mediterranean Sea and settled into its ecosystem, pufferfish, known for their poisonous characteristics, are of special concern [178]. TTX appearance is a rather recent phenomenon for Mediterranean countries as the silver stripe blaasop (*L. sceleratus* (Gmelin, 1789)) invaded the Mediterranean Sea. *L. sceleratus* has had negative ecological, economical, and health effects in the Mediterranean basin [179]. This species has expanded rapidly and has now reached Western Mediterranean waters. After the pufferfish invasion in the Mediterranean, TTX poisoning cases began to appear in Israel [17,40,180], Lebanon [181], Spain [24], and Turkey [42,44,182]. Two cases of poisoning were also reported in Italy, but these cases originated from imported Monkfish [183,184].

**Table 7:** Global cases of TTX intoxication in humans reported in the literature.

Country	Number of Total Cases	Number of Ratal Cases	Source of Intoxication	Case Year	Reference
Australia	11	-	Pufferfish	2001–2002	[167]
	7	-	Toadfish		[168]
Bangladesh	141	17	Pufferfish	2008	[185]
	37	8	Pufferfish	2002	[186]
	53		Pufferfish	2001-2006	[187]
	36	7	Pufferfish (*Takifugu oblongus*)	2002	[163]
	6	-	Pufferfish	2005	[161]
	8	5	Pufferfish	1998	[162]
	11	-	Pufferfish	2014	[109]
Brazil	11	-	Pufferfish	-	[170]
	27	2	Pufferfish	1984–2009	[171]
	1	1	Pufferfish	-	[172]
Cambodia	57	9	Pufferfish	2003–2007	[153]
China	30		Gastropod, *Zeuxis samiplicutus*	2000 (Zheijiang)	[188]
	40	8	Gastropod, *Nassarius* spp.	1991–2003 (Lianyungang)	[189]
	309	16	Gastropod, *Nassarius* spp.	1977–2001 (Zhoushan)	[190]
	59	18	Gastropod, *Nassarius* spp.	1985–2000 (Ningbo)	[191]
	150	6	Gastropod, *Nassarius* spp.	2002–2005 (Fujian)	[147]
	22		Goby fish	2012	[146]
Egypt	59	14	Pufferfish	2008–2010	[176]
India	8	-	Pufferfish	2007 (Burla)	[166]
Indonesia	6	6	Pufferfish	2001	[164]
Israel	13	-	Pufferfish	2005	[17]
	1	-	Pufferfish	2012	[39]
	2	-	Pufferfish	2008	[180]
Italy	10	-	Monkfish (*Lophius piscatorius*) imported from Taiwan	1977 (Roma)	[183]
	3		Monkfish (*Lophius piscatorius*) imported from Taiwan	1978 (Pavia)	[184]
Japan	1	-	Thread-sail filefish	2008	[68]
	1	-	Marine snail	2007	[68]
	5	-	Marine snail, *Babylonia japonica*	1957	[192]
	1	-	Marine snail, *C. saulie*	1979	[193]
	1	1	Pufferfish	-	[194]
	1322	265	Pufferfish	1965–2002	[110]
	632	19	Pufferfish	2003–2020	[145]
Lebanon	1	-	Pufferfish	2008	[181]
Madagascar	17	5	Pufferfish	1993–1998	[174]
Malaysia	6	3	Horseshoe crab	2011	[159]
	3	-	Pufferfish	2008	[160]
Mexico	18	2	Pufferfish	1970–2000	[172]
Morocco	3	1	Pufferfish	-	[177]
New Caledonia	1	-	Diodontidae	2007	[169]
Oman	5	1	Pufferfish	2018	[173]
Reunion Island	10	-	Pufferfish	2013	[175]
Singapore	1	-	Pufferfish	-	[165]
South Korea	7	-	Pufferfish	2002–2011	[155]
	41	-	Pufferfish	2004–2010 (Seul)	[157]
	40	-	Pufferfish	1995–1998	[154]
	111	30	Pufferfish	1991–2002 (Busan, Gyeongsangnam-do, Jeollanam-do Jeju-do)	[156]
Spain	1	-	Trumpet shellfish (*Charonia sauliae*)	2007	[24]
Thailand	55	-	Pufferfish	-	[149]
	8	-	Pufferfish (*Tetraodon fangi*)	1988	[195]
Taiwan	2		*Papillosus alectrion* and *Nassarius gruneri*	2002	[151]
	192	22	Pufferfish Gastropod (snail) Goby	1988–2011	[152]
	2	-	Blue-lined octopus *Hapalochlaena fasciata*	2010	[90]
Turkey	7	3	Pufferfish	2020–2021	[42,44,182]
USA	2	-	Pufferfish (imported)	2014 (Minnesota)	[196]
	5	-	Pufferfish (imported)	1996, 2006 (California)	[197]
	1	-	Pufferfish (imported)	1986 (Hawaii)	[125]
	2	-	Pufferfish (imported)	2007 (Chicago)	[197]
	1	-	Pufferfish (imported)	2007 (New Jersey)	[197]
Vietnam	87	2	Blue-lined octopus *Hapalochlaena fasciata*	2004	[130]
	7	3	Gastropod, *Nassarius* spp.	2006–2007	[158]

Where the USA is concerned, it appears that poisoning cases are generally caused by imported pufferfish. As such, a relevant incident involving 10 cases of intoxication, where the patients eventually recovered, was caused by mislabeling [125,196,197].

## 6. Treatment of TTX Intoxication in Human

The high fatality rates and lack of any specific antidote are persisting challenges for TTX intoxication cases. Taking necessary precautions against poisoning is the only way to avoid the risks, potentially including death, associated with TTX poisoning cases [198]. In fact, the treatment of human TTX intoxication is largely symptomatic and involves supportive care, including respiratory support measures, until TTX is excreted in the urine. It has been shown indeed that supportive care, including emesis, gastric lavage and respiratory support, and fluid replacement, reduced deaths in TTX intoxication cases [48,68,125,186].

Firstly, if spontaneous vomiting has not already occurred, the toxin should be expelled from the body by inducing vomiting by emetic agents, such as apomorphine, to reduce exposure to unabsorbed TTX [125]. Gastric decontamination should ideally be done if the patient is brought to the hospital within 60 min after TTX poisoning. For this purpose, gastric lavage, especially with sodium bicarbonate solution (2%), followed by activated charcoal, is recommended, since TTX is less stable in an alkaline medium [125]. In the early stages of TTX intoxication, activated charcoal can be administered orally to victims to prevent the gastric absorption of the toxin [199]. Secondly, as the two main causes of death are respiratory arrest and severe hypotension, respiration should be secured, and oxygenation should be provided. In cases of respiratory distress or failure, oxygen and other respiratory support, including endotracheal intubation, are often required to maintain cardiovascular function until the toxin is completely eliminated from the body [142]. As such, in the Thailand TTX poisoning outbreak, patients were treated with endotracheal intubation and mechanical ventilation. As a result, 239 (97.5%) of 245 patients recovered completely, 1 patient (0.4%) had brain damage, and 5 patients (2%) died due to the intoxication [127]. Similarly, in Israel, TTX intoxication cases recovered within 4 days by providing respiratory support during the poisoning outbreak [17]. Further treatment including fluid and electrolyte replacement could be used to reduce resulting fluid loss, to induce urinary excretion of the toxin and to enhance cardiac output and systemic vascular resistance [142]. In addition, hemodialysis may be useful, especially in patients with renal disease or dysfunction. Considering the clinical signs of various seafood poisonings, PSTs intoxication is the most difficult to distinguish from TTX poisoning [200]. Detailed and accurate information for the history of exposure is critical for differential diagnosis since the organisms harboring other neurotoxins are usually different from TTX-containing species [201]. A complete physical examination, including a comprehensive neurological examination, electrophysiological studies, and analytical techniques could be useful in making a differential diagnosis of TTX poisoning; routine laboratory tests are not helpful [135].

An experimental animal study showed that amphetamine, phenylephrine, and norepinephrine are the most effective agents for the treatment of serious hypotension in TTX intoxication, possibly due to their direct or indirect adrenergic effects [133]. However, some investigators suggested the administration of dopamine as the first-line inotropic agent [125]. Some researchers have also proposed using atropine in patients with bradycardia, but its clinical effect is controversial [140,142]. Atropine is not routinely required, as bradycardia is not a common serious problem in human TTX intoxication [134].

Moreover, certain drugs could be useful in relieving the various symptoms, including restoring motor activity associated with TTX intoxication. Some researchers report that administration of the anticholinesterases, such as edrophonium and neostigmine, enhance the recovery of motor power and markedly reduce paresthesia and numbness by increasing the acetylcholine level at the neuromuscular junction [39,142,202,203]. However, other investigators did not support these reports [122,124,140] since TTX probably does not act on the motor end-plate until its concentration reaches a high level [204], indicating that anticholinesterase drugs are not likely to be useful for the treatment of TTX intoxication [122]. The efficacy of neostigmine in 37 TTX intoxication cases has been reviewed by Liu et al., who concluded that the current literature was insufficient to provide an evidence base for or against the use of neostigmine in patients with TTX-associated respiratory failure [205].

Hemodialysis could be a potentially useful approach, especially in patients with renal disease or renal dysfunction. However, there is very limited information in the literature regarding the effectiveness of dialysis in the treatment of human TTX intoxications. It has been reported that hemodialysis applied 21 h after TTX exposure was effective in a uremic patient with serious neurologic dysfunction [206,207]. Recently, hemodialysis following TTX poisoning 12 h after exposure was reviewed in two patients from Oman, who recovered, but the report did not provide enough evidence to support this therapeutic approach in human TTX intoxication cases [174]. In the same report, it was suggested that, to prove the efficiency of hemodialysis, the toxin in the removal of ultrafiltrate could be determined. However, other investigators have indicated that hemodialysis may not be an effective treatment since the toxin has low water solubility [122,208].

For a long time, several experimental studies have been carried out to find a specific antidote and to develop effective treatments against human TTX intoxication. Antiserum and monoclonal antibodies against TTX have been developed and tested successfully in experimental animals [209,210]. Various monoclonal antibodies have been developed against TTX [211,212] and tetrodonic acid [213]; however, none of these were shown to be effective in vivo following TTX exposure. It has been also reported that a polyclonal rabbit anti-TTX antibody was effective for protecting mice from lethal TTX exposure [214]. In another study, monoclonal antibodies protected the animals from TTX intoxication by neutralizing the toxin, resulting in 100% survival [215]. Furthermore, Rivera et al. developed a specific monoclonal antibody against TTX and reported that this antibody was effective for protecting mice from lethal TTX exposure [210]. On the other hand, an effective TTX-specific vaccine was developed, and it was demonstrated to successfully protect animals from haptenic TTX by enhancing humoral immune response [115,216,217]. Although these products have a therapeutic potential for the treatment of TTX intoxication, further investigations are needed to provide enough evidence for their efficacy in real human cases.

## 7. Therapeutic Use of TTX in Medicine

Various marine natural compounds have the potential to be used as medicines in the treatment of various diseases, further to their use as experimental tools or food supplements [2,218,219,220,221,222]. In this context, potent marine toxins, such as TTX, have received particular attention from researchers in the last three decades and gained importance as experimental tools because of their specific targets and mode of their pharmacological activity [223]. Due to the particular significance of pufferfish in Japanese culture, scientific research on the pharmacological and toxicological effects of pufferfish or TTX have been carried out mostly by Japanese researchers for a long period of time.

Following the discovery of the TTX molecule’s ability to block Na^+^ ion channels in nerve cells, scientific studies mostly focused on the cellular and molecular mechanisms of TTX [224]. As such, despite its potent neurotoxicity, TTX was considered possible be used in medicine as an analgesic to treat various types of pain, due to its blocking of specific Na^+^ ion channels and paralyzing effect. TTX targets specific Na^+^ ion channels and has been used mainly as a popular chemical tool or a product in various scientific studies of neurophysiological and pharmacological processes mediated by those ion channels [2,3,98]. The limitations for its medical use are related to its toxic effects; nevertheless, its potent pharmacological activity shown in several clinical trials and experimental animal models supports its rational use for therapeutic purposes. Although there are many investigations on the medical use of TTX in the literature, most of these studies focused on its analgesic and local anesthetic properties, due to its blocking of very specific Na^+^ ion channels.

Therapeutic use of TTX is mainly based on blockade of very specific voltage-gated Na^+^ ion channels with a high degree of selectivity and, thus, suppresses action potentials in axons and reduces ectopic peripheral nerve activity [225]. TTX displays analgesic activity by inhibiting the initiation and conduction of action potentials and, consequently, blocking nerve transmission in the peripheral nervous system [226]. TTX was used as an analgesic agent for the treatment of neuropathic and rheumatic pains in the early 20th century in Japan [58]. TTX has also been used as an analgesic agent in terminal cancer patients in China [223]. Furthermore, in various studies, summarized in Table 8, researchers have conducted some preclinical and clinical studies for the use of subtoxic doses of TTX as an effective analgesic agent in the treatment of various intense pains, such as in severe cancer patients [227,228,229,230]. In addition, Campos-Ríos et al. [231] recently emphasized that TTX is a potential analgesic that can be used to treat visceral pain, especially painful gastrointestinal conditions.

A Canadian pharmaceutical company (WEX Pharmaceuticals, Inc., Vancouver, Canada) has developed a pharmaceutical form containing TTX for subcutaneous injection (Halneuron; Tectin; Tetrodin; Tocudin) as an analgesic in advanced cancer patients to reduce the intense pain as an alternative to narcotics and opioid pain medication and the treatment of opiate addiction. An earlier open-label, multi-center clinic trial indicated that two or three times daily TTX administration for 4 days caused a clinically significant reduction in pain intensity, and relief of pain persisted for up to two weeks in 17 out of 31 treatments in patients with severe cancer-related pain [232]. In another multi-center clinic trial performed in Canada, TTX was administered at a dose of 30 μg intramuscularly twice daily for 4 days to cancer patients [230]. In this trial, according to only pain score assessment, TTX did not provide clinically significant analgesia in a heavily pre-treated cohort of cancer patients with moderate to severe pain. However, according to an analysis of secondary endpoints, and an exploratory post hoc analysis, the authors suggested that TTX may potentially relieve moderate to severe pain in cancer patients, and often for prolonged periods following treatment, with mild side effects. In a further clinic trial for evaluation of long-term safety and efficacy properties of TTX, a sustained analgesic effect with usually acceptable toxicity was observed in patients with cancer-related pain following subcutaneous administration of 30 μg TTX twice daily for 4 days [230]. Similarly, later clinical trials also indicated that TTX may provide clinically meaningful analgesia with acceptable side effects at the same dose and treatment course in cancer patients [227,228]. Besides these, in vivo and in vitro genotoxicity assays indicated that TTX did not have any genotoxic potential for patients [233], and this could be an advantage for its use as an analgesic agent in medicine [2].

**Table 8:** Therapeutic use of TTX in clinical treatments.

Study Direction	Number of Participants	Study Design	Dose and Exposure	Outcome and Results	Reference
Cancer related pain	24	An open-label, multi-dose study	TTX 15 to 90 μg daily, administered intramuscularly in divided doses, over four days	TTX was overall safe. It effectively relieved severe, treatment-resistant cancer pain in the majority of patients and often for prolonged periods after treatment	[232]
	77	A randomized, double blind, parallel design multi-center study	TTX (30 μg, bid) was administered subcutaneously for 4 days	This study suggested TTX may potentially relieve moderate to severe, treatment-resistant cancer pain in a large proportion of patients, and often for prolonged periods following treatment, but further study is warranted using a composite primary endpoint	[229]
	41	A multi-center open-label longitudinal and efficacy trial	TTX (30 μg) was administered subcutaneously twice daily for 4 days	Long-term treatment with TTX is associated with acceptable toxicity and, in a substantial minority of patients, resulted in a sustained analgesic effect	[230]
	149	A multi-center, randomized, double-blind, placebo-controlled, parallel-design trial	TTX (30 μg) was administered subcutaneously twice daily for four consecutive days	TTX may provide clinically meaningful analgesia for patients who have persistent moderate to severe cancer pain despite best analgesic care	[228]
	125	A randomized, double blind, placebo controlled, parallel dose comparison study	TTX (7.5, 15, and 30 μg/kg BID and 30 μg/kg QD) administered as subcutaneous injections for 4 days	This study suggests the TTX 30 µg b.i.d. regimen is well tolerated with promising early efficacy data	[227]
Heroin dependence	45	Double blind, placebo-controlled	TTX (5 µg or 10 µg) administered intramuscularly	Low-dose TTX is acutely effective in reducing cue-induced increases in heroin craving and associated anxiety	[234]
	216	A multi-center, randomized, double-blind, placebo-controlled	TTX (5 µg or 10 µg) administered intramuscularly	TTX significantly reduced withdrawal symptoms by day 3 compared with placebo, and there was no significant difference in the incidence of adverse events in study groups	[235]

Moreover, the potential effects of TTX against drug addiction behaviors have been investigated in human patients. Intramuscular administration of low dose TTX (5 µg or 10 µg) was found to be effective in reducing cue-induced increases in heroin craving and associated anxiety with no sign of systemic side effects [234]. In addition, a significant reduction of heroin withdrawal symptoms by TTX has been shown in patients with a diagnosis of heroin dependence at dosages of 5 and 10 μg three times a day [235]. In addition, it has been indicated that the intramuscular pre-treatment of TTX substantially prevented morphine withdrawal symptoms in mice and rats without any systemic adverse effects [236]. Although its mechanism of action has not yet been fully elucidated, it was suggested that TTX may be an alternative drug in the treatment of opiate addiction [234].

The potential analgesic effects of TTX have been investigated in various experimental studies at subtoxic doses in rodent models, summarized in Table 9. Nieto et al. [237] reported that the subcutaneous injection of low doses TTX (1, 3, or 6 µg/kg) could be useful to prevent and treat paclitaxel-induced neuropathic pain in mice. Besides, the analgesic effects of subcutaneous TTX injection have been indicated by the formalin test and the partial ligation of the sciatic nerve (Seltzer’s model) in rats without causing any adverse effects [238]. Kayser et al. [239] reported that subcutaneous injection of TTX (0.3, 1, 3, or 6 μg/kg,) into the back displayed antihyperalgesic effects and decreased pain-related behaviors in rats with injured sciatic nerve through mechanisms that involve complex interactions with endogenous opioid system. In addition, it was shown that local injection of TTX (0.03–1 μg) into the gastrocnemius muscle provided effective analgesia in rats with persistent muscle pain produced by carrageenan injection [240]. Gonzalez-Cano et al. [241] concluded that subcutaneous administration of TTX decreased pain-related behaviors and reversed the mechanical hyperalgesia in the colon and peritoneum induced by capsaicin and cyclophosphamide injections in viscera-specific mouse models, respectively. Furthermore, TTX decreased thermal hyperalgesia and mechanical allodynia using a full-thickness thermal injury model in rats following subcutaneous administration at a dose of 8 μg/kg [242]. These animal studies demonstrated that subtoxic doses of TTX could be used as an analgesic drug in neuropathic and inflammatory pain with lower adverse effects. STX displays a similar mode of action and analgesic properties to TTX and induces anesthesia or prolongs the anesthetic effect of local anesthetics in combination treatments [243]. However, its systemic toxicity limits its clinical use as a therapeutic agent. To prevent its toxicity for the treatment of joint pain and intractable localized pain, the preparation of STX by means of microencapsulation in liposomes has been proposed [244]. It has been indicated that this liposomal formulation produced a prolonged nerve blockade in a neuropathic pain model in rats without any toxicity [245]. Moreover, other studies showed that an N-1 hydroxylated STX analogue, neoSTX, displayed efficacy for both acute and chronic pain treatment in rats [246] and in patients with somatic [247] and visceral pain [248] without any adverse effects. 

**Table 9:** Summary of the experimental animal studies on analgesic effects of TTX.

Experimental animals	Model-Technique	Dose and Exposure	Outcome and Results	Reference
Wistar rats Swiss and Webster mice	The formalin test and to partial ligation of the sciatic nerve (Seltzer’s model)	TTX (0.3, 1, 3, or 6 μg/kg) administered subcutaneously 30 min before the formalin test	TTX decreased pain behavior in the formalin test at the highest dose and in the writhing test at 3 and 6 mg/kg. It also reduced mechanical allodynia and thermal hyperalgesia with an ED_50_ of 1.08 and 0.62 mg/kg without any adverse effects, respectively	[238]
CD-1 mice	Neuropathic pain induced by paclitaxel	TTX (1, 3, or 6 µg/kg) administered subcutaneously	Low doses of TTX can be useful to prevent and treat paclitaxel-induced neuropathic pain, and that TTX-sensitive subtypes of sodium channels play a role in the pathogenesis of chemotherapy-induced neuropathic pain	[237]
Male Sprague–Dawley rats	Chronic unilateral constriction injury to either the sciatic nerve or the infraorbital nerve	TTX (0.3, 1, 3, or 6 μg/kg) administered subcutaneously into the back	TTX alleviates pain-related behaviors in sciatic nerve-lesioned rats through mechanisms that involve complex interactions with endogenous opioid systems	[240]
Male Sprague-Dawley rats	Full thickness thermal injury (FTTI) model	TTX (8 μg/kg) administered subcutaneously	TTX reduced thermal hyperalgesia and mechanical allodynia	[242]
Adult male Sprague–Dawley rats	Carrageenan (k-carrageenan, 1% in NaCl 0.9%) was injected into the belly of the gastrocnemius muscle,	Local injection of TTX (0.03–1 μg) into the gastrocnemius muscle	TTX displays important analgesic effects on rat models of persistent muscle pain, without interfering with the nociceptor function to signal for further potentially harmful stimuli	[240]
Adult wild-type Nav1.7 knockout (KO-Nav1.7) mice	Viscero-specific mouse models of chemical stimulation of the colon (intracolonic instillation of capsaicin and mustard oil) and intraperitoneal cyclophosphamide-induced cystitis	TTX (3 and 6 μg/kg) administered subcutaneously	This study suggests that blockade of TTX-sensitive sodium channels, but not Na_v_1.7 subtype alone, by systemic administration of TTX might be a potential therapeutic strategy for the treatment of visceral pain	[241]

Intensive research has been carried out for many years to develop a local anesthetic or a local anesthetic-sustained release system that can provide long-lasting peripheral nerve blockade with minimal systemic or local toxicity. Various scientific studies have indicated that TTX, a specific Na^+^ ion channel blocker, produces a potent and long-lasting local anesthesia and causes minimal local and systemic adverse effects at safe doses (Table 10). Topical injections of TTX with epinephrine generated an effective and prolonged local anesthesia of the sciatic nerve in rats and provided reversible blocks that lasted over 13 h at a dose of 11.5 µM [249]. TTX injected with either bupivacaine or epinephrine, resulted in prolonged nerve blockade, with less toxicity compared to bupivacaine administration alone [250]. Besides, co-encapsulation of TTX (75 mg, 0.05%, *w*/*w*) in controlled release devices containing dexamethasone and bupivacaine generated effective and long-lasting topical nerve blocks in rats [251]. Similarly, polymer TTX conjugates (1.0–80.0 µg) produced a range of prolonged local anesthesia of nerve block in rats, from several hours to 3 days and causes minimal local and systemic toxic effects [252]. TTX has some advantages, such as not having direct side effects, such as myocardial depression and poorly crossing the blood brain-barrier, compared to conventional local anesthetic agents [253].

The effect of TTX on topical ocular anesthesia has been also investigated experimentally in animal models (Table 10). These studies indicated that TTX applied topically in the eye provided an effective and prolonged topical anesthesia for pain control in surgery procedures in rabbits [254,255]. Besides, Green et al. [256] showed that topical corneal application of TTX (1 mM, 10 μL) in 0.9% saline significantly alleviated photophobia in rats with corneal injury. They suggested that TTX could be used as an effective therapeutic option to reduce the symptoms of photophobia that occurs after ocular surgery and other clinical diseases. It has also been demonstrated that TTX was effective in mitigating ischemic damages caused by occlusion of hippocampus vessels [257] and those caused by exposure to veratridine in neurons of the cerebrum and hippocampus [258] in rats. In addition, previous experimental studies (Table 10) in rats showed that focal injection of TTX was effective in reducing damage at the injury site and attenuated neurological deficits and tissue loss following spinal cord injury [19,259,260]. Moreover, the inhibition of amyloid beta (A4) precursor protein (APP) by TTX (1 μM) has been demonstrated by a western blot technique in rat hippocampal slices to investigate neuronal activity for regulation processing in the mammalian brain [261]. Furthermore, an in vitro study indicated that stimulus train-evoked seizures were blocked after local injection of 50 μM TTX in rat hippocampal slices [262]. TTX blocked the electrographic seizures when applied in the perfusion medium with lower concentrations of (5, 10, or 20 nM). TTX was also found effective to prevent post-traumatic epileptogenesis [263]. Evoked epileptiform field potentials were observed in the injured cortex, and thin sheets of Elvax polymer containing TTX implanted over lesions were effective in decreasing evoked epileptiform potentials. Furthermore, Kitamura et al. [264] showed that TTX decreased the expression of activity-dependent three genes that are likely to be key factors in the regulation of synaptic plasticity in cerebral cortical cells from E18 rat embryos.

The antitumor effect of TTX has been investigated in experimental animals with tumor and in the in vitro studies with cancer cell lines summarized in Table 10 and Table 11, respectively. It has been reported that TTX obtained from the skin of the masked pufferfish (*Arothron diadematus*) was applied to mice with Ehrlich Ascite Carcinoma, resulting in an increase in survival and a decrease in the number of tumor cells [265,266]. Besides, the inhibitory effect of TTX has been shown on prostate cancer using in vitro cell culture model with human glioma cell lines ((HTB-138) or MAT-LyLu and the AT-2 cell lines [267,268,269]. The extracts of three strains of TTX-producing bacteria (*Bacillus sp.*, *Kytococcus sedentarius*, and *Cellulomonas fimi*) isolated from *Arothron hispidus* type pufferfish caught from the southeast coast of India were intraperitoneally injected into mice with leukemia, and growth inhibitory effects were observed on the muscle and leukemia cell lines [270]. Similarly, the anticancer activities of TTX, obtained from 3 three species of TTX-producing bacteria (*Vibrio alginolyticus*, *Microbacterium arabinogalactanolyticum*, and *Serratia marcescens*) were investigated in vitro using SW480 and SW620 colorectal carcinoma cell lines. The results of the study indicated that TTX has a substantial inhibitory effect on both cell lines [271]. The researchers suggested that TTX-producing bacteria isolated from pufferfish can be used to develop potential anti-tumor compounds.

**Table 10:** Summary of the experimental animal studies on other therapeutic effects of TTX other than its analgesic effect.

Experimental Animals	Pharmacological Activity	Model- Technique	Dose and Exposure	Outcome and Results	Reference
Adult male Sprague-Dawley rats	Local anesthesia	Sciatic Blockade Technique	Co-administration of capsaicin and TTX-loaded liposomes	The combined delivery of capsaicin and TTX using a sustained-release system can achieve prolonged duration local anesthesia without detectable toxicity	[272]
		Sciatic Blockade Technique	TTX (15.95 mg/L) and bupivacaine (4442 mg/L) or epinephrine (10.08 mg/L)	TTX injected with either bupivacaine or epinephrine, results in prolonged nerve blockade, with myotoxicity that is no worse and perhaps less than that from bupivacaine	[250]
		Sciatic Blockade Technique Modified hotplate test Weight-bearing test	Polymer-TTX conjugates (1–80 μg)	1.0–80.0 µg of TTX released from these polymers produced a range of durations of nerve block, from several h to 3 days, with minimal systemic or local toxicity	[252]
		Sciatic Blockade Technique	Rats received sciatic nerve blocks with 75 mg of microspheres containing 0.05% TTX, 50% bupivacaine and/or 0.05% dexamethasone	Co-encapsulation of TTX in controlled release devices containing bupivacaine and dexamethasone resulted in very prolonged nerve blocks	[251]
		Sciatic Blockade Technique	TTX [0.3 mL, 10 μM-20 μM (3.19 6.38 mg/L)] with and without epinephrine (10.08 mg/L) or bupivacaine (4442 mg/L)	Bupivacaine increased the local anesthetic potency of tetrodotoxin, reduced its systemic toxicity, and, when co-injected subcutaneously, increased the median lethal dose from 13.95 to 15.23 μg/kg	[249]
		Sciatic Blockade Technique and Neurobehavioral Assessment of Nerve Blockade	TTX [0.3 mL of 3.19 mg/L (0.96 μg dose)] with bupivacaine 0.25% (2.5 mg/mL) with or without epinephrine 5 µg/mL	Blocks containing bupivacaine 0.25% with TTX 3.19 mg/L and epinephrine 5 µg/mL were prolonged by roughly 3-fold compared to blocks with bupivacaine 0.25% plain (P < 0.001) or bupivacaine 0.25% with epinephrine 5 µg/mL (P < 0.001)	[253]
New Zealand White rabbit	Ocular local anesthesia	TTX was applied into the inferior conjunctival cul-de-sac of the right eye	A 40 TTX (32, 319 or 3190 mg/L) or proparacaine 0.5%	TTX is a long-acting topical anesthetic in the rabbit cornea (At a dose of 3190 mg/L, TTX produced an anesthesia up to 8 h)	[254]
Adult male Sprague Dawley rats	Attenuation of photophobia	Photophobia by corneal de-epithelialization injury	Topical corneal application of TTX (319 mg/L, 10 μL) in 0.9% saline	TTX markedly attenuated photophobia in rats with corneal injury TTX may be an effective therapeutic option to reduce the symptoms of photophobia that occurs after photorefractive keratectomy and other clinical diseases	[256]
Female Sprague-Dawley rats	Ameliorate effect by local blockade on spinal cord injury	A laminectomy was performed	Microinjection of TTX (0.5 μL of 95.7 mg/L–47.8 ng dose)	The results demonstrate that TTX preserves axons from loss after spinal cord injury	[19]
		A laminectomy at the T8 level exposed a 2.8-mm-diameter circle of dura	TTX (47.85-319 ng/L) 0.5 to 2 μL microinjected into the injury site	The TTX group exhibited a significantly enhanced recovery of coordinated hindlimb functions, more normal hindlimb reflexes, and earlier establishment of a reflex bladder	[260]
Male Sprague–Dawley rats	Anticonvulsant	Cortical injury in a model of chronic epileptogenesis TTX-impregnated Elvax Neocortical slices Behavioral observations	TTX/Elvax 20 mg/g. The slices incubated with 1.6–15.96 µg and 319 µg/L TTX	The findings indicated that TTX prevents posttraumatic epileptogenesis in rats in a model of chronic epileptogenesis	[263]
Adult female Swiss albino mice injected tumor cell line	Anticancer	Ehrlich ascites carcinoma-EAC)	TTX (1/20 of the LD_50_) administrated intraperitoneally after 10 days into EAC mice	Treatment with TTX caused a significant decrease in the mean tumor weight and an increase in the cumulative mean survival time when compared with EAC group	[265]
Adult Swiss female albino mice		Ehrlich Ascite Carcinoma (EAC)	TTX extracted from the fish skin and applied as a dose of 1/10 and the 1/20 of the LD_50_	Exposure to TTX caused the rate of cell division to be reduced greatly, especially in the first 6 days post-treatment. The authors suggested that the reduction in cell number is probably due to increased apoptosis	[266]
Male albino mice		Mouse muscle cell line (L929) and leukemia cell line	TTX intraperitoneally administered with 0.25, 0.50, 0.75, and 1.0 mL and dissolved at 5 mg/ml	TTX inhibited the growing of the muscle and leukemia cell lines. It was suggested that TTX can be used to develop anti-tumor compounds	[270]

Recently, Law et al. [273] showed that TTX is a potent active compound against SARS-CoV-2 according to a ligad-based approach using a Ligand Scout 4.3 software and ligand-based pharmacophore model generator. However, further in vivo and in vitro studies would be needed to confirm this possible antiviral activity.

**Table 11:** Summary of the in vitro studies on the therapeutic effects of TTX.

Study Technique	Pharmacological Activity	Model-Technique	Dose and Exposure	Outcome and Results	Reference
Cell culture	Anticancer	Human glioma cell lines (HTB-138)	Cell cultures exposed with TTX concentrations of 3.19 and 6.38 mg/L for a period of 24 and 48 h	TTX exert the inhibitory effect on the invasion of metastatic prostate cancer	[274]
		Metastatic MAT-LyLu Cell Line of Rat Prostate Cancer	TTX at different concentration between 0.32 µg and 319 µg/L	TTX inhibits (IC_50_: 5.75 μg/L) invasiveness of metastatic prostate cancer	[275]
		MAT-LyLu and AT-2 prostatic carcinoma cell lines	TTX at 319 μg/L	Migration of the MAT-LyLu cell line was reduced significantly by TTX (at 319 μg/L); in contrast, there was no effect on AT-2 cell motility	[267]
		MAT-LyLu and AT-2 prostatic carcinoma cell lines	Incubation of TTX at 1.91 mg/L for 24 h	TTX produced significant changed the morphology of MAT-LyLu cancer cell	[268]
Cell culture	Neuroprotective effect	Rat cerebellar neurons	TTX 1.6–31.9 µg/L	TTX protected cultured neurons from veratridine-induced toxicity and could be use in treatment of ischemic neuronal injury by preventing excessive neuronal depolarizations.	[258]
Male Sprague-Dawley rat hippocampal slices	Anticonvulsant effect	Hippocampal slices blocked stimulus train-evoked electrographic seizures (EGSs)	Localized injection of TTX	Stimulus train-evoked seizures were blocked after localized injection of 15.95 mg/L TTX in rat hippocampal slices. Low concentrations of TTX (1.6, 3.19, or 6.38 µg/L) in the perfusion medium blocked EGSs without decreasing the amplitude of extracellular responses to single stimuli	[262]
Ligand-based pharmacophore modeling and Ligand Scout 4.3 software.	Antiviral activity against SARS-CoV-2.	Modeling	Structure-based pharmacophore modeling	TTX is a potent active compound against SARS-CoV-2 according to ligand-based approach	[273]

## 8. Analytical Methods for the Detection and Quantification of TTXs

Several methods have been described for the detection and quantification of TTXs. In what follows, bioassays, cell-based assays (CBAs), immunoassays, aptamer-based assays, biosensors, and instrumental analysis techniques for TTXs are summarized. Further details can be found in the mentioned bibliographic references and in more specific methodological reviews [3,9,27,276,277,278,279].

### 8.1. Bioassays

As for many other toxins and compounds in general, bioassays were the first methods used for the detection of TTXs. However, due to their low specificity and ethical concerns, they have been replaced by other analytical methodologies. Nevertheless, the mouse bioassay (MBA) is sometimes still used due to the toxicological information that provides [18,77,91].

### 8.2. Cell-Based Assays and Biosensors

As in saxitoxin (STX) and STX analogues, differently than ciguatoxins (CTXs), TTX and TTX analogues block voltage-gated sodium channels (VGSCs) of cells in a close state. This blocking affects the passive influx of sodium ions, resulting in numbness, respiratory paralysis, mild gastrointestinal effects, and even death in humans that have consumed TTX-contaminated food. This mechanism of action is also the basis of most CBAs, where TTXs rescue cells from the effect of ouabain (which inhibits the Na^+^/K^+^-ATPase pump) and veratridine (which blocks VGSCs in an open state and promotes sodium influx into the cells), and increase their viability. CBAs for TTXs may not be selective enough, since other VGSC blockers, such as STXs or gonyautoxins, may co-occur in a sample. Nevertheless, they are very useful as screening tools, to detect unknown toxic compounds, and for the establishment of toxicity equivalency factors (TEFs). TEFs are the ratio of the toxicity of one compound to the toxicity of the reference compound within the same toxin group and are used in instrumental analysis to provide an estimation of the overall toxicity of a sample.

There are several methods to detect cell viability in CBA for TTXs, including light microscopy [280], where morphological changes of cells are measured, colorimetry with several dyes, such as Neutral red [281], or tetrazolium salts [282,283], and fluorescence with propidium iodide [284,285]. Other more sophisticated techniques measure cell action potentials. Whereas patch clamp electrophysiology monitors voltage across the membrane of individual cells [284], the use of multi-electrode arrays (MEAs) for the immobilization of neurons in not invasive and allows measurement of extracellular potentials or impedance changes due to cell attachment/detachment [286,287]. These techniques are difficult to implement in monitoring programs and are usually limited to electrophysiological research studies. Nevertheless, efforts have been made to facilitate high throughput detection, for example using 48- [288] and 96-well [289] sensor plates with embedded electrodes, which showed limits of detection (LODs) of 0.3 and 89 ng/mL, respectively. It is also interesting the capability of cell-based biosensors to respond to different marine toxins simultaneously, such as the system developed for the detection for TTX, STX, (brevetoxin-3) PbTX-3, Pacific ciguatoxin-1 (P-CTX-1), palytoxin (PlTX), and domoic acid (DA), where the LOD for TTX was 0.3 ng/mL [288]. Although these toxins have different mechanisms of action, local field potentials from cells can be recorded for all of them. This cell-based array was successfully applied to the analysis of a spiked pufferfish sample.

### 8.3. Immunoassays, Immunosensors and Immunostrips

Due to the small size of TTX (319 Da), the production of antibodies has not been an easy task. Nevertheless, several antibodies have been obtained using carrier proteins, which need to be different to the coating agents in the immunoassays to avoid non-specific adsorptions [290,291,292,293,294]. Recombinant antibody fragments have also been synthesized through phage display technology and exploited in immunoassays [289]. Although some competition direct formats have been developed [295], immunoassays for TTXs have commonly adopted competition indirect formats with immobilized TTX. The method used to immobilize TTX on microtiter wells is crucial since its antigenic site needs to be accessible. In this direction, the use of long chemical linkers instead of protein carriers for TTX tethering in an ordered and well-oriented way has been proposed [6]. This immunoassay, which attained an LOD of 2.28 ng/mL, was used in the establishment of cross-reactivity factors (CRFs) of several TTX analogues, obtained from a pool of toxic puffer fish, in their biding with the monoclonal antibody. The response of the immunoassay to TTX analogues was evaluated and compared to that of parent TTX, and the same was performed with a Surface Plasmon Resonance (SPR) biosensor where TTX had been immobilized with another linking strategy. CRFs between the immunoassay and the SPR biosensor were different for the different TTX analogues, demonstrating that the TTX immobilization is crucial as also are the assay configuration and detection method. This immunoassay was successfully applied to the analysis of pufferfish from Greece [6] and Spain [296] and, with a small variation (short linkers instead of long linkers), to shellfish [297] and urine [296]. The binding of polyclonal antibodies, recently produced, against TTX analogues from toxic puffer fish [298] but also marine ribbon worms [299], has also been evaluated. It is interesting to note that the group of the haptenic antigen used in the link with the carrier protein for the antibody production has a crucial effect on the cross-reactivity of the different TTX analogues. In fact, the cross-reactivity with other toxins, such as STXs, may also be associated to the chemical synthesis and, therefore, has to be carefully taken into account when interpreting the results from an immunochemical assay [9].

As in immunoassays, the antigen immobilization is also crucial in biosensors. Efforts have been made to optimize TTX immobilization protocols in optical SPR immunosensors with the purpose to maximize specific binding, while minimizing non-specific binding [300,301], and guarantee the applicability of the developed tools to the analysis of different samples, such as pufferfish, gastropods, urine, milk, and apple juice [301,302,303,304]. Apart from the conventional indirect formats, direct detection has also been achieved in SPR, providing lower LODs, shorter analysis times, decreased use of reagents, and improved confidence [305]. Planar waveguide, another optical technique, has also been exploited in the development of immunosensors for the detection of TTXs in pufferfish [306].

Several electrochemical immunosensors have been developed for TTX. The first biosensor relied on the use of protein conjugates for electrode surface coating [307]. In this work, alkaline phosphatase, the enzyme used as a label, was conjugated to the anti-TTX antibody. This strategy shortens the assay time, but stability of the conjugates still needs to be addressed. As in immunoassays, other approaches pursued TTX tethering through chemical linkers. This is especially important in electrochemical biosensors, where proteins conjugates can hinder electron transfer. That is the case of the work described by Reverté and co-workers [308], where TTX was immobilized on gold electrode arrays and the subsequent biosensor, which attained an LOD of 2.6 ng/mL, was applied to the analysis of pufferfish. More recently, the use of magnetic beads (MBs) as TTX immobilization supports has been demonstrated to be a very good strategy to remove or at least decrease matrix effects in both immunoassays [309] and immunosensors [310]. The use of MBs in suspension certainly favors the antibody/antigen interaction and makes the washing steps more efficient, which result in lower LODs (0.5 and 1.2 ng/mL, respectively). Removal of matrix effects is especially key in the analysis of shellfish samples, where low LODs are pursued, since the European Food Safety Authority (EFSA) is proposing a guidance threshold of 44 µg TTX equiv./kg [12], much lower than the 2 mg TTX equiv./kg edible portion used in Japan as the acceptance criterion to consider pufferfish safe for consumption [162].

Immunostrip tests with antibodies for the detection of TTX have also been developed [311,312,313]. All these lateral flow assays (LFAs) use gold nanoparticles as a label. These LFAs are especially useful for rapid analysis of samples in an easy way. Although these tools are not quantitative, as they rely on eye detection, the simplicity and rapidity, as well as the lack of needing instrumentation, make them advantageous for in situ analysis. Screening of pufferfish samples [312], as well as clams and other fish species, such as crucian [313], with immunostrip tests has been successfully demonstrated. To avoid subjectivity and provide more precise measurements, LFAs can be coupled to portable readers. This is the case of the works where fluorescence quenching was used as a detection method [101,314].

### 8.4. Aptamer-Based Assays and Aptasensors

Aptamers are short oligonucleotide sequences with high affinity for their target analytes, produced using a methodology based on the systematic evolution of ligands by exponential enrichment (SELEX). As for antibodies, the production of aptamers for TTX is not an easy task. Nevertheless, several aptamers have been recently described. The first one was obtained in 2012 and exploited in a fluorescence assay [315]. Afterwards, this aptamer was used to develop a direct impedimetric aptasensor [316]. Although the selectivity against other toxins was not checked and natural samples were not analyzed, results were promising, obtaining an LOD of 0.199 ng/mL. This aptamer was also used in the development of another fluorescence assay that used berberine as a fluorescent reporter [317], attaining an LOD of 0.024 ng/mL. In this case, the selectivity was checked. However, since the authors evaluated the applicability of the assay to the analysis of spiked human body serum samples, the analytes chosen for the cross-reactivity study were possible coexisting substances in such a matrix, i.e., Na^+^, K^+^, Cl^−^, CO_3_^2−^, PO_4_^3−^, cysteine, glutathione, uric acid, lysine, glucose, tryptophan, serine, and aspartic acid. In their subsequent work, where an even lower LOD was obtained (0.004 ng/mL), spiked pufferfish samples were also analyzed [318].

Another anti-TTX aptamer was produced by Gu and coworkers [319], which developed a multiplex SELEX to simultaneously produce the aptamers for STX and DA, based on the use of magnetic reduced graphene oxide as a solid support for the separation of affinity oligonucleotides from non-affinity ones. The corresponding fluorescence assay provided an LOD of 1.21 ng/mL and excellent recovery values in the analysis of spiked clam samples. This aptamer was also exploited in a sophisticated fluorescent assay [320]. In this work, the aptamer was immobilized on MBs for the subsequent competition with free TTX and an oligonucleotide strand complementary to the aptamer. The complementary oligonucleotide remaining after competition was detected using a triple cycle amplification combining strand displacement amplification with catalytic hairpin assembly. The system, which attained an extremely LOD of 0.265 pg/mL, showed no cross-reactivity against other marine toxins and provided excellent recovery values in the analysis of spiked shellfish samples.

Recently, a new aptamer has been produced using a capture-SELEX process, in which the oligonucleotide library was immobilized on magnetic beads, and exploited in a hybrid antibody-aptamer assay [321]. This is one of the few examples of sandwich assay for small molecules. The hybrid format is very attractive because combines the advantages of both types of biorecognition elements, an aptamer and an antibody, providing the appropriate sensitivity/specificity. The assay shown an LOD of 310 pg/mL and no cross-reactivity with DA, okadaic acid (OA), nor STX, the latter sometimes simultaneously present in puffer fish or shellfish. The assay was applied to the analysis of a *L. sceleratus* puffer fish individual, providing TTX contents similar to those obtained with other analytical techniques.

### 8.5. Instrumental Analysis Techniques

The first chemical methods for the detection of TTXs were based on high performance liquid chromatography with post-column fluorescence derivatization (HPLC-FLD) [322,323,324]. These methodologies were applied to the analysis of toads [325,326] and newts [327,328], after proper sample clean-up.

In more recent works, fluorescence has been replaced by mass spectrometry (MS), which provides higher specificity and improves the detection of some TTX analogues with low fluorescence intensity, such as 5-deoxyTTX and 11-deoxyTTX [329]. The LOD for TTX on the LC/MS in the selected ion monitoring (SIM) mode via electrospray ionization (ESI) was 223 pg, approximately twice of that of the LC-FLD (127 pg). The tandem mass spectrometry (MS/MS) scan of the fragment ions of eight TTXs arising from the molecular ions provided characteristic spectra. This method was applied to the analysis of puffer fish samples, with recoveries between 77.7–80.7% and an LOD of 0.1 mg/kg [330]. Since the structures of TTX and its analogues are very similar and, therefore, their relative response factors in SIM mode of LC/MS analysis are very close to each other, TTX has also been used as an internal standard for quantification of TTXs analogues [331]. The use of a hydrophilic interaction chromatography (HILIC) column coupled to MS detection decreased the LODs 3 times compared with previous LC-MS methods [332,333] and was applied to the analysis of pufferfish [334,335,336] and gastropods [337,338]. Methods for the analysis of TTX using triple quadrupole LC/MS-MS instrumentation and multiple reaction-monitoring (MRM) have also been developed and applied to the analysis of bivalves [79,339]. High resolution mass spectrometry (HRMS) has allowed the identification of a new analogue of TTX, 5,11-dideoxyTTX, in pufferfish and flatworm by comparison of retention times and fragmentation patterns with those of totally synthesized [62]. This study has contributed to understand metabolic processes, since this compound is supported to be a biosynthetic intermediate of TTX in stepwise oxidation process from 5,6,11-trideoxyTTX to TTX.

It is also important to mention that LC-MS/MS has also been applied to the detection and quantification of TTXs in human body fluids, such as urine and plasma, to confirm TTX poisoning [276,296,340,341,342].

Finally, the co-occurrence of STXs and TTXs in several organisms has led to the development of instrumental analysis methods for the detection of both groups of toxins [343,344,345,346].

### 8.6. Advantages and Limitations of the Different Analytical Methods

There is not a gold standard method for the detection of TTX in a sample. Many times, the combination of methods is the best choice because different methods provide complementary information that contribute to better know the TTXs content of a sample. However, this is not always possible, and the amount of sample, analysis time, and cost are important parameters in the choice.

MBA and CBAs provide a composite toxicological response and are very useful for the detection of unknown toxic TTX analogues, which may not be targeted in instrumental analysis. MBA, although very cheap, has specificity and ethical concerns. The main limitation of the CBAs is that harmonization is difficult, much more than, for example, in immunoassays. Additionally, high amounts of sample and long analysis times are required. The use of living material in CBAs implies special care in the laboratory and work under strict conditions. It may cause differences in sensitivity, which, on the other hand, could be overcome by constructing daily calibration curves. One important advantage is the medium cost, which makes it preferred in low-income countries.

Biochemical assays and the corresponding biosensors, i.e., those based on antibodies and aptamers, are highly interesting because they are specific, sensitive, and usually easy to apply. Ideally, antibodies and aptamers should recognize those TTX analogues that are toxic, and with CRFs that are equivalent to the TEFs, but this may not be always the case. Nevertheless, although only a few CRFs have been established with immunoassays/immunosensors and none with aptamer-based assays/sensors, these biomolecules use to target the parent TTX, which usually is the most abundant and toxic analogue. Compared to antibodies, aptamers do not involve animal experimentation and do not suffer from batch-to-batch variation. Additionally, they can easily be modified and tuned to be adapted to specific assay formats. However, the strict conditions necessary for the binding step make it difficult to apply them in the analysis of natural samples, where components of the extracts may interfere in the assay. In general, biosensors have already been demonstrated to be successful in the analysis of natural samples (mainly immunosensors). The medium cost and convenient assay time make them highly promising tools. However, their commercialization is still a pending task. Miniaturization, simplification of the transducers and portability of the devices are key. In this direction, immunostrip tests, although they do not provide a quantitative measurement, should be cheaper and easier to deploy and implement. 

Instrumental analysis is highly sensitive and specific but requires sophisticated and expensive equipment and skilled users. They are also limited by the availability of standards and reference materials. The high cost hampers its implementation in some developing countries. Nevertheless, their use as a confirmatory method is undeniable. Additionally, it can provide information about the TTXs profile of a sample and help to understand the metabolic processes.

## 9. Concluding Remarks and Future Orientations

The research progress in the TTX field during the last few years, in all aspects analyzed in the present review, is highly intertwined with the health concerns arising from the increasing records of TTX presence in edible marine organisms, as well as its expansion in new latitudes. Efforts towards development of validated analytical methods, studies attempting to elucidate TTX origin and sources in nature, as well as the level of human exposure to TTX, together with new data on its pharmacological properties and potential beneficial effects for human health have been substantial, indicating the ongoing character of the TTX issue at a global level and the necessity for effective risk assessment and management to counteract the problems arising from this fascinating toxin group. In this context, certain remarks are provided as “food for thought” to trigger further research in the TTX field and potentially instigate scientific dialogue on future approaches on TTX regulatory management.

### 9.1. Origin and Sources

Although numerous recent works have added new insights regarding the sources and origin of TTX in nature and its accumulation in edible marine organisms, the exact mechanisms remain largely unknown, thus hindering the introduction of appropriate risk management measures, such as health warnings [8]. As such, targeted research is required to elucidate these mechanisms, as well as TTX kinetics in marine organisms, especially as regards clarification of between-species differences in accumulation and depuration levels and rates. Significant uncertainty is still present concerning TTX producing organisms, as currently available scientific evidence is sometimes contradictory. In this context, more intense investigations on the TTX-producing potential of specific bacterial species are required, firstly involving their isolation and culture and subsequent explication of any influencing factors. On the other hand, possible synergies among different types of microorganisms, such as symbiotic relationships between microalgae and bacteria, have also attracted attention, however, with quite inconclusive outcome, probably owing to strain-specific differences in the studied cases.

### 9.2. Occurrence and Distribution of TTXs in Potentially Edible Aquatic Organisms 

The data provided within Table 1, Table 2, Table 3 and Table 4 indicate an increased frequency of TTX detection in the recent years, accompanied by a gradual expansion of the number of new species and locations where TTX is being encountered. On the other hand, the levels found in these novel occurrences, with the exception of pufferfish and certain gastropods, seem to be generally lower than those historically found. This may be attributed to several factors. For instance, older records were mostly associated with poisoning incidents, triggering the relevant investigations to identify the causative agents, whereas sensitivity and selectivity limitations of determination methods may also have masked the potential presence of lower TTX concentrations. Consequently, the optimized performance of modern analytical methods, together with more intense monitoring programs for marine toxins’ presence, often extended to incorporate those considered emerging due to their suspected interconnection with climate change effects, may have also significantly contributed to this rise of TTX reporting, often as an accidental finding. Nevertheless, the possibility of an increasing prevalence cannot be excluded, hence multiplying concerns on potential risks to human health. The recent introduction of the EFSA proposed provisional limit [12] for bivalve mollusks and gastropods, set at 44 μg TTX/kg shellfish meat, was an initial step towards counteracting hazards derived by consumers’ exposure to TTX. The concentration levels encountered in mollusks and gastropods often exceed the EFSA limit, sometimes more than 10-fold, yet very limited associated poisoning incidents have been reported. On the other hand, in edible parts of these organisms, only few specimens exceeding the Japanese limit of 2000 μg/kg have been recorded. This may indicate that the EFSA proposed level may be too low and should be revisited, in light of the latest occurrence data, to reevaluate its suitability for combining human health protection with ensuring the viability of the shellfish industry [47]. In this context, obtaining further occurrence data in a variety of edible species and locations should be prioritized to allow for more robust risk assessment approaches and to reduce uncertainty as regards to the necessity of introducing regulatory measures for TTX risk management.

### 9.3. Mode of Action and Toxicity of TTX 

Although TTX’s mode of action has been long known and animal acute toxicity data are largely available, recent works providing insights on the potential chronic effects of TTX exposure [347,348] indicate that the proposed EFSA limit is appropriate to prevent adverse effects on humans associated with daily TTX exposure. Moreover, a synergistic effect was discovered upon combined administration of TTX and STX in mice, with the lethality of the former increased in the presence of STX, indicating that these two toxin groups should be considered in conjunction when evaluating risks and establishing potential regulatory approaches. Further research should investigate the extent of such interactions, taking into account the common mode of action of TTX and STX, as well as other potential synergies with other toxin groups. In this direction, a retrospective analysis of past intoxication incidents involving edible marine organisms where both TTX and STX were detected could also provide valuable insight on the in vivo synergies of these two toxin groups and the effectiveness of a consolidated risk management strategy, taking into account that STX is already regulated. 

### 9.4. Treatment of TTX Intoxications and Therapeutic Use of TTX 

The discovery of an efficacious antidote to counteract TTX-induced toxic consequences in humans has long been pursued by the scientific community. Although significant progress has been made towards achieving this target, encompassing the development of specific antibodies and vaccines and subsequent testing in animal models, more solid evidence is required to confirm their potential for use in human intoxication cases. On the other hand, exploitation of TTX’s pharmacological properties for therapeutic uses, such as alleviating human discomfort or inducing anesthesia, although still at its infancy, constitutes a promising field for future research. Such applications are considered of particular interest, given the plethora and recent range expansion of TTX-containing marine organisms, such as pufferfish, in new localities previously perceived as TTX-free. For instance, the currently widespread over-presence of *L. sceleratus* in the Mediterranean Sea could be partially mitigated in the future by means of commercial exploitation of the species as a TTX source for pharmaceutical and/or medical use [72]. In this context, further studies on potential TTX medical/pharmacological applications should be prioritized, whereas establishing an appropriate regulatory framework for allowing invasive pufferfish commercialization outside the alimentation scope must be considered in parallel, to remove current legislative obstacles in this direction.

### 9.5. Analytical Methods for the Detection and Quantification of TTXs 

The last decade has been signified by tremendous advancements in the development of analytical methods for TTX determination, mostly associated with technological improvements in analytical equipment and the pressure to move away from bioassays for specificity and ethical reasons, as well as the increased scientific interest in this toxin group. Nevertheless, several gaps are still identified in the analytical front, which, in turn, render difficult the adoption of routine monitoring approaches. A few certified reference materials (CRMs) for TTX are now commercially available in solution form, but respective matrix counterparts are still missing. Furthermore, the current CRMs provide a limited coverage as regards TTX analogues, whereas toxic equivalence factors for the latter are still highly uncertain. In this context, further research aiming to acquire more solid scientific information on TTX analogues’ presence and relative potencies is a prerequisite. On the other hand, recently developed multi-toxin LC-MS/MS methods able to simultaneously detect and quantify STXs and TTXs [55,343] may constitute promising options to facilitate incorporation of TTX into routine monitoring schemes without considerable additional burdens in cost or time. Such alternative approaches may prove highly applicable, taking into account the potential for risk management consolidation for these two toxin groups.
